# Recent Developments in the Detection of Organic Contaminants Using Molecularly Imprinted Polymers Combined with Various Analytical Techniques

**DOI:** 10.3390/polym15193868

**Published:** 2023-09-24

**Authors:** Tomasz Nazim, Aleksandra Lusina, Michał Cegłowski

**Affiliations:** Faculty of Chemistry, Adam Mickiewicz University in Poznań, Uniwersytetu Poznańskiego 8, 61-614 Poznań, Poland; tomasz.nazim@amu.edu.pl (T.N.); aleksandra.lusina@amu.edu.pl (A.L.)

**Keywords:** molecularly imprinted polymers (MIPs), MIPs sensors, analytical techniques

## Abstract

Molecularly imprinted polymers (MIPs) encompass a diverse array of polymeric matrices that exhibit the unique capacity to selectively identify a designated template molecule through specific chemical moieties. Thanks to their pivotal attributes, including exceptional selectivity, extended shelf stability, and other distinct characteristics, this class of compounds has garnered interest in the development of highly responsive sensor systems. As a result, the incorporation of MIPs in crafting distinctive sensors and analytical procedures tailored for specific analytes across various domains has increasingly become a common practice within contemporary analytical chemistry. Furthermore, the range of polymers amenable to MIP formulation significantly influences the potential utilization of both conventional and innovative analytical methodologies. This versatility expands the array of possibilities in which MIP-based sensing can be employed in recognition systems. The following review summarizes the notable progress achieved within the preceding seven-year period in employing MIP-based sensing techniques for analyte determination.

## 1. Introduction

Molecularly imprinted polymers (MIPs) represent a remarkable class of polymeric systems engineered to recognize specific target analytes. Achieving selective analyte recognition involves preparing MIP materials in the presence of designated analyte molecules during the polymerization step, which plays a pivotal role in shaping the interactions at this stage. These interactions, which are influenced by both the chemical formula of the MIP side chain and the analyte, encompass various bonding forces, such as hydrogen bonds, van der Waals forces, and π-π interactions. Following the template removal, distinct MIPs’ cavities are formed, constituting the foundation of the material’s selective recognition properties. The efficiency of these cavities is closely linked to the shape, size, and functional groups of the incorporated compounds, allowing for modulation based on their chemical formulas [1]. Enhancing the recognition properties is an essential pursuit, and this can be achieved by increasing the number and strength of interactions. Thus, the synthesis of MIPs should prioritize the incorporation of a higher number of functional groups. However, it is important to consider that an excessive abundance of functional groups may unexpectedly compromise the recognition efficiency [2]. Consequently, the successful design and development of novel MIP materials necessitate a thoughtful selection of compounds to shape the final interactions precisely.

Benefitting from their valuable properties, such as high selectivity, shelf stability, recovery, and repeatability, MIPs find extensive applications in real-time analysis and point-of-care testing [3]. Notably, MIPs exhibit the ability to determine complex samples, rendering them suitable for biological sample treatment. The growing demand for stable and cost-effective materials in the realm of smart device design has sparked a burgeoning interest in utilizing MIPs as molecular recognition particles in sensors, leveraging their selective recognition capabilities. Various systems have been harnessed for targeted analyte determination, wherein MIPs play a pivotal role. For instance, the rebinding of the analyte to the MIPs’ cavity can be detected through changes in the refractive index in surface plasmon resonance sensors, electrochemical impedance spectroscopy, or other innovative techniques [4].

While MIPs offer numerous advantages, they also present several prevalent challenges, including issues related to binding site heterogeneity, template bleeding, and the optimization of synthesis processes for targeted templates [5]. After the removal of the template molecules, it is essential for the molecular cavities to maintain a harmonious balance between structural rigidity and flexibility. This equilibrium is pivotal in facilitating swift and dynamic interactions between the polymer matrix and the analyte, thereby ensuring efficient adsorption and desorption processes. The optimization process thus necessitates a comprehensive comprehension of the chemical equilibrium states, principles of molecular recognition, thermodynamics, and polymer chemistry in order to craft a polymer with the desired properties [6]. Due to the fact that the most extensively researched method for MIPs’ synthesis is free radical polymerization, in this process, the pivotal roles are played by the functional monomers and cross-linkers. Typically sourced from the reservoir of chemical compounds encompassing acrylates, methacrylates, or vinyl species, these constituents are instrumental in shaping the structural and functional attributes of MIPs [7]. This leads to various additional drawbacks, with the most prominent ones being a low binding capacity, template residue, and limited compatibility with aqueous solutions [8]. The resolution to addressing these drawbacks lies in exploring alternative MIP synthesis methods. These include techniques such as living/controlled radical polymerization, surface imprinting, nano-imprinting, or the exploration of new polymeric systems amenable to molecular imprinting [9].

Thanks to the versatility of the used MIP materials, when combined with a diverse array of analytical techniques, it becomes possible to develop specialized sensors capable of effectively detecting an extensive range of analytes without limitations. As a consequence, there has been extensive research into the integration of MIPs into sensor prototypes, leading to the commercialization of MIP-based sensors [10]. The literature thus reports the use of commercial MIP-based sensors for real-life applications (acoustic wave devices [11,12], optical sensors [13], medical treatment [14,15], and others [10]).

This review explores diverse techniques employing MIPs as selective recognition sensors for compound determination. The subsequent sections delve into several chosen analytical techniques, showcasing the versatility of MIPs in determining a wide array of analytes. This review encapsulates the significant advancements made in recent years in the application of MIPs for selective analyte determination. Hence, this review highlights its novelty by presenting a wide range of detailed analytical techniques, including both well-established methods and innovations reported within the last seven years. These methodologies can be synergistically employed in conjunction with MIP materials to facilitate the direct analysis of contaminants. This review provides a comprehensive overview of various analytical techniques that utilize molecularly imprinted polymers for the detection of organic contaminants. In contrast, other review articles offer more specific details, concentrating on particular analytical methods [16,17] or specific substances of interest [18,19].

## 2. Chromatographic Methods

### 2.1. High-Performance Liquid Chromatography

High-performance liquid chromatography (HPLC) was first developed in the early 1960s and has become a widely used analytical technique. HPLC falls under the category of liquid chromatography, where the separation occurs between a mobile phase (the solvent) and a stationary phase (the column packing). The process involves passing the sample mixture under a given pressure through an HPLC column packed with the stationary phase. As the sample travels through the column, each component of the mixture interacts differently with the stationary and mobile phases, leading to differential retention times. The components that have stronger interactions with the stationary phase will be retained longer in the column and take more time to elute from the column. On the other hand, components that have weaker interactions with the stationary phase will elute faster and have shorter retention times. This principle enables the detection and quantification of compounds in the investigated sample. Detection and quantification are done using a detector, such as a UV-Vis detector, fluorescence detector, or mass spectrometer. By analyzing the separated compounds’ retention times and peak areas, the HPLC system can identify and quantify the individual components present in the studied mixture [20,21,22].

There are four main modes of HPLC separation, depending on the nature of the stationary phase [20,22]: adsorption chromatography, partition chromatography, ion exchange, and size exclusion chromatography.

The application of HPLC has expanded beyond traditional liquid chromatography, and newer variations, such as high-performance thin-layer chromatography (HPTLC), ultra-high-performance liquid chromatography (UHPLC), and two-dimensional chromatography, have emerged to address specific analytical challenges. With the continuous progress in HPLC methodologies and its compatibility with various detection techniques, such as UV-Vis, fluorescence, and mass spectrometry, this analytical tool continues to play an important role in analytical chemistry [23,24,25].

The HPLC method is commonly used for studying MIPs to assess their selectivity and binding capabilities toward specific molecules.

Chromatographic measurements involving MIPs can occur in both offline and online modes [26,27]. In addition, molecularly imprinted materials can be used in various forms and shapes. For example, MIPs can be used as different-shaped small particles [28], monolithic columns [29,30], or microspheres [31].

The offline approach relies on the equilibrium binding analysis, which helps to compare the performances of different MIPs. It removes time-related effects and provides information about the affinity and binding capacity of the MIP. In this process, a known amount of MIP is mixed with a measured quantity of the analyte in a specific volume. The mixture is agitated until equilibrium between the solid MIP and the liquid phase containing the analyte is reached. After that, the liquid phase is separated, and the amount of free analyte is determined. The difference in analyte concentration from the initial incubation is considered to have bound to the MIP. This data is used to create an adsorption isotherm, which shows the equilibrium concentration of the analyte associated with each phase, either in the solution or adsorbed on the MIP [32].

In addition to the described indirect analysis of MIPs by studying analyte-depleted solutions (which can also be examined using other methods such as electrochemical techniques or mass spectrometry), MIPs can be employed as sorbents for HPLC analyses. In HPLC, the proper pretreatment of samples is essential in analyzing real samples due to their complex compositions and sometimes low concentrations of analytes. Among the methods enabling analyte preconcentration, solid-phase extraction (SPE) is one of the most commonly used techniques. Selecting an efficient sorbent material allows for the reduction in solvent usage and research costs. It enables to reduce matrix effects, standardizes sample variability, enhances sensitivity, and purifies the samples. Above all, employing SPE facilitates enhanced measurement sensitivity, separation of desired analytes from complex matrices, and removal of interfering substances. An SPE technique that utilizes MIPs as the sorbent is usually called molecularly imprinted solid-phase extraction (MISPE) [33,34,35,36,37].

**Figure 1:**
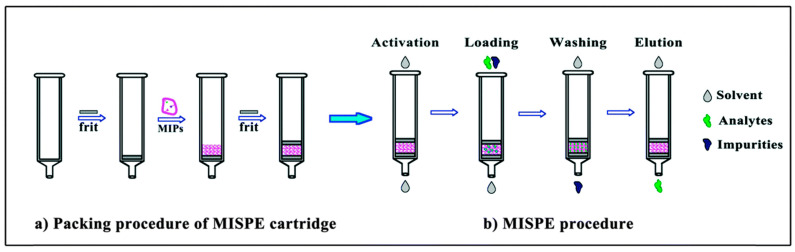
A simplified diagram illustrating offline MISPE preparation and the actual procedure. Reprinted from [7]. Copyright (2023), with permission from Elsevier (or applicable society copyright owner).

**Figure 2:**
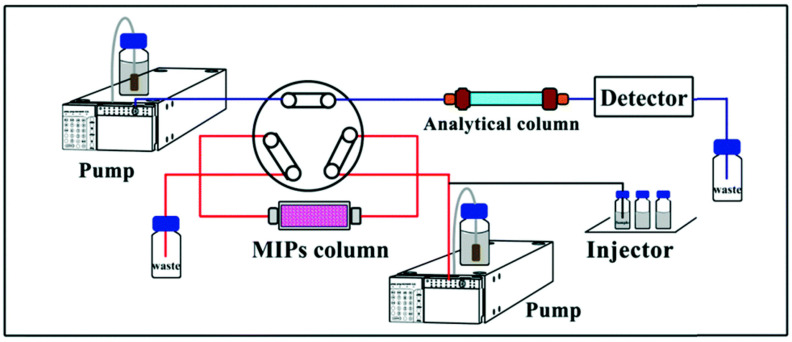
A simplified diagram illustrating online MISPE preparation and the actual procedure. Reprinted from [7]. Copyright (2023), with permission from Elsevier (or applicable society copyright owner).

MISPE is a valuable method that utilizes MIPs as solid materials to selectively enrich and extract target compounds from complex samples before subjecting them to HPLC analysis. In this technique, an HPLC cartridge is packed with MIPs that have been tailored with specific binding sites for the target compounds. The sample containing the target compounds of interest is introduced into the MISPE column. As the sample elutes through the column, the target compounds selectively interact and bind to the MIPs due to the pre-designed recognition sites. This retention allows for the removal of unwanted interfering compounds. Subsequently, the column is washed with an appropriate solvent to remove any weakly bound components. The target compounds are then eluted from the column using another solvent, and the eluate is collected for subsequent HPLC analysis. Depending on which stage the extracted analyte solutions will be analyzed, one can distinguish between offline or online techniques. In the offline technique, analyte-enriched solutions after elution upon leaving the cartridge are transferred to the chromatograph and subsequently analyzed (Figure 1). In the online mode, the cartridge is integrated into the instrument, resulting in the analyte solutions being analyzed on the spot (Figure 2). The selective enrichment provided by MISPE enhances the sensitivity and precision of the HPLC analysis, particularly for trace-level analytes in complex matrices [27,35,36,37].

Numerous scientific studies utilized the HPLC method to analyze designed MIP properties [38,39,40,41,42,43,44]. In these studies, HPLC was used to analyze the concentration of the analyte before and after the adsorption process, allowing for the characterization of the process and determination of its kinetics, adsorption capacity, selectivity, and recovery rate.

In [38], a molecularly imprinted polymer was synthesized for the selective extraction of fenoprofen (a nonsteroidal anti-inflammatory drug) in aqueous environmental samples. The MIP exhibited a Langmuir isotherm and pseudo-second-order kinetics, indicating monolayer chemisorption on the surface. The MISPE technique was employed to isolate fenoprofen from aqueous samples selectively. Offline HPLC analysis of fenoprofen in wastewater using the MIP as a sorbent provided a detection limit of 0.64 ng mL^−1^ (2.64 nM). The limit of detection (LOD) for the pure solvent was determined at 1.90 nM and a recovery of 99.6% was achieved under optimal conditions.

Other investigated studies included sulpiride (an antipsychotic medication) as the template molecule [40]. The synthesized MIP exhibited high adsorption properties with an imprinting factor of 5.36 and a maximum adsorption capacity of 61.13 μmol g^−1^. Furthermore, the MIP was successfully applied in MISPE for sulpiride extraction from mixed solutions and serum samples, achieving extraction recoveries ranging from 81.57% to 86.63%. The MIP showed promising potential as an efficient sorbent for selective isolation of sulpiride in biosample analysis.

The following study also investigated other drugs from the sulfonamide group [44]. This study focused on a multi-template surface MIP prepared with and without prepolymerization to detect six sulfonamides in real water samples simultaneously. The MIP in this approach was combined with magnetic graphene oxide coated with mesoporous silica. The magnetic properties exhibited by the synthesized material facilitated separation from the solution. The MIP with prepolymerization exhibited higher sulfonamide adsorption capacities, leading to an efficient SPE. The offline method demonstrated excellent applicability for detecting trace sulfonamides in real water samples. The MIP with prepolymerization achieved spiked recoveries ranging from 73.34% to 99.43% and relative standard deviations (RSDs) between 2.28% and 7.77%. In comparison, the MIP without prepolymerization showed recoveries of 87.37% to 102.34%, with RSDs between 3.18% and 6.49% for the six sulfonamides during SPE.

Another example of an investigation of magnetoactive polymers was proposed by Fu et al. [39]. In this study, magnetic molecularly imprinted polymers (MMIPs) were analyzed for the concentration of zearalenone (a mycotoxin) in cereals. MMIPs exhibited good adsorption properties, with a maximum adsorption capacity of 13.90 mg g^−1^ for zearalenone. The selective binding ability of zearalenone was confirmed through kinetic and Scatchard analysis. The developed MMIPs showed a maximum adsorption capacity of 13.90 mg g^−1^ for zearalenone, with a limit of detection and limit of quantification of 0.4 ng kg^−1^ and 0.9 ng kg^−1^, respectively, demonstrating their effectiveness for zearalenone determination in cereals.

Selective and efficient magnetic molecularly imprinted polymers were also developed to extract trace residuals of the synthetic estrogen dienestrol (a nonsteroidal estrogen) in seawater [43]. The MMIPs exhibited core–shell structures with large binding capacities, showing nearly three times higher adsorption capacity for the analyte than magnetic molecularly non-imprinted polymers. The method allowed for the enrichment of dienestrol from spiked seawater samples with satisfactory recoveries (87.3–96.4%) and low RSD values (2.03–5.18%). The LOD and limit of quantification (LOQ) were 0.16 µg L^−1^ and 0.52 µg L^−1^, respectively, and the MMIPs showed no significant deterioration in adsorption capacity after six rounds of regeneration. The HPLC method proved to be effective for analyzing dienestrol in seawater without time-consuming procedures, addressing the low tolerance of traditional adsorption materials to high-salinity matrices.

The approach involving the combination of polydopamine and magnetic nanoparticles, along with the creation of a molecular imprint using 17β-estradiol as the template, was implemented in the study by Wang et al. [45]. In their work, the authors achieved “daisy-like” Fe_3_O_4_ nanoparticles via chemical etching. The chemically etched nanomaterials possessed reduced weight compared with their spherical precursors and provided an abundance of imprinted sites, which possibly enhanced the interaction with the template. The magnetic properties of nanomaterials significantly improved the separation process. Consequently, these resulting nanomaterials exhibited rapid adsorption equilibrium (10 min). Moreover, the adsorption capacity reached 83.03 mg g^−1^. In real sample analysis, the authors achieved an LOD value of 0.28 ng mL^−1^ (1.03 nM).

In another study undertaken by the same authors [46], a strategy involving the synthesis of polydopamine with molecular imprinting in the presence of SiO_2_ and Fe_3_O_4_ was employed to create hollow-surface molecularly imprinted polymers. Through the etching of SiO_2_, the authors obtained a unique porous surface, which increased the available molecular imprinting sites for tetracycline molecules. This resulted in a high adsorption capacity of 70.23 mg g^−1^. In environmental samples, using milk as the matrix, the authors achieved an LOD value of 0.83 ng mL^−1^ (1.87 nM).

An interesting approach was utilized by Sun et al. [42]. This study introduces a method involving a dummy molecularly imprinted polymer to detect climbazole (an antifungal agent). The MIP was synthesized using miconazole as a dummy template. The obtained material exhibited remarkable selectivity and binding capacity for the template, which was demonstrated via HPLC and equilibrium binding experiments. An important outcome of this study was the successful development of a robust method that combined dummy MISPE with HPLC to selectively enrich climbazole from environmental water samples using MIP as the sorbent. The optimization of DMISPE conditions, including sample loading pH/volume, selective washing, and elution solvents, contributed to the method’s efficacy. The method’s performance was assessed using spiked samples at various concentration levels (0.2, 1.0, and 5.0 μg L^–1^), yielding favorable recoveries (82.3–96.2%) and consistent repeatability (RSDs 0.6–4.9%, *n* = 5). Notably, the LOD was determined to be 0.012 μg L^–1^, affirming the method’s sensitivity. It is worth mentioning that the materials were investigated online, where the column containing the MIP was integrated into the device and the solution after elution containing the analyte was analyzed without additional processing.

In the literature, there are also numerous examples of analyzing organic compounds using the combined method of HPLC and MS. LC-MS combines the separation capabilities of liquid chromatography with the high sensitivity and specificity of mass spectrometry. It is widely used in MIP research to identify and quantify target analytes, especially in complex matrices, such as biological or environmental samples. The LC part of the technique separates the analytes based on their chemical properties, while the MS part detects and characterizes the separated analytes based on their mass-to-charge ratio. LC-MS provides valuable information on the selectivity and sensitivity of MIPs in real sample analysis. LC-MS/MS, also known as tandem mass spectrometry, further enhances the capabilities of LC-MS by providing increased sensitivity and selectivity through multiple stages of sample fragmentation analysis, making it a powerful tool for MIP research in complex sample analysis [47,48,49,50].

The study by [51] is an exemplary application of tandem mass spectrometry as a detection technique, specifically utilizing LC-MS/MS to analyze and quantify chloramphenicol (an antibiotic) in milk samples. The MIP exhibited significant specific recognition for chloramphenicol and was successfully applied for solid-phase extraction in milk samples. The method coupled with LC-MS/MS showed excellent recoveries (96.04–108.68%) and precision (RSDs < 7.97%) for chloramphenicol in milk samples. The LOD and LOQ were determined as 0.02 μg L^−1^ and 0.08 μg L^−1^, respectively.

In a similar study, roxithromycin (an antibiotic) molecularly imprinted polymers were investigated [52]. The paper presents a simple and reliable method for determining roxithromycin in human plasma using MMIPs as sorbents. The MMIPs exhibited high affinity and selectivity for roxithromycin, allowing for a single-step extraction process with easy separation using an external magnet. LC-MS/MS was employed for the subsequent analysis of the eluted analyte. The LOD and LOQ achieved were 3.8 ng mL^−1^ and 9.8 ng mL^−1^, respectively, indicating the method’s sensitivity. Moreover, the recoveries of roxithromycin from human plasma samples ranged from 86.5% to 91.5%, demonstrating the proposed technique’s high extraction efficiency and accuracy.

In [53], an MIP was synthesized to extract selected pharmaceuticals belonging to five different classes from surface water samples. The pharmaceuticals targeted in this study included an antiretroviral (nevirapine), an antidepressant (venlafaxine), a muscle relaxant (methocarbamol), an anticonvulsant (carbamazepine), and a cardiac stimulant (etilefrine). In this research, MIP was developed for the selective extraction of pharmaceuticals from surface water samples, showing high affinity and efficiency for five different classes of drugs. The MIP-based solid-phase extraction coupled with LC-MS analysis exhibited promising results, with detections ranging from 0.03 to 0.31 ng mL^−1^ and recoveries ranging from 43% to 69% in dam water samples.

The study by [54] serves as an example of the application of HPLC and MIPs in the food industry. A selective analytical method combining microwave-assisted extraction, MISPE, and LC-MS was developed to determine bisphenol A in canned food. The method demonstrated high precision and a low detection limit and was successfully applied to commercial canned samples with bisphenol concentrations ranging from 7.3 to 42.3 ng g^−1^. The aim was to efficiently extract the analyte from complex food matrices using a selective extraction solvent and enhance the sample clean-up for accurate analysis. In addition to the standard procedure, the LOD of the method was also tested in various canned food products, including tuna, pineapple, and mushrooms. The achieved values ranged from 0.3 to 1.4 ng g^−1^.

The developed analytical procedure using HPLC and MIPs as solid phase extraction SPE can be applied to analyze various dyes, including textile dyes [55].

### 2.2. Gas Chromatography

Gas chromatography (GC) is another powerful chromatographic technique for analyzing volatile and thermally stable compounds. In the context of MIPs, GC (gas chromatography) is particularly useful for studying analytes with low molecular weights. The technique relies on separating analytes based on their volatility and interactions with the stationary phase. GC can provide valuable information about the specificity and efficiency of MIPs in the analysis of small organic compounds. The principle of GC is similar to the previously described HPLC method, but there are several fundamental differences. First, the main difference lies in the different mobile phases; in GC, it is a carrier gas, such as helium or nitrogen, while in HPLC, it is a liquid solvent. GC also requires strict temperature control, unlike HPLC. GC is commonly used for analyzing volatile organic compounds and specific small molecules. Another difference appears in the stationary phase; in GC, the stationary phase can also be a liquid [56,57].

While MIPs integrated with HPLC and GC can be used for selective analyte separation and preconcentration, the choice of technique depends on the specific application and the nature of the target analytes. HPLC-MIPs are more commonly used for liquid samples, while GC-MIPs are often employed for vapor-phase or headspace analysis. Similar to HPLC, the conducting of MISPE via GC can also be achieved offline and online.

The study by [58] is one example of utilizing GC in conjunction with MIPs to develop a rapid and sensitive method for detecting multi-pesticide residues in vegetables. The researchers synthesized an MIP using O,O′-dimethylthiophosphoryl chloride as the template and evaluated its selectivity for five organophosphorus pesticides. The MIP was applied in offline MISPE coupled with GC to efficiently detect trace pesticide residues in leaf lettuce and cucumber samples. In the results, the synthesized MIP exhibited high selectivity for the target pesticides, with adsorption capacities significantly greater than non-imprinted polymer NIP, confirming its specific recognition ability. The enrichment factor ranged from 36 to 452 for the five pesticides under optimal solid-phase extraction conditions, ensuring efficient detection of trace levels of pesticide residues. The obtained LODs in this study ranged from 0.15 to 0.89 μg L^−1^.

The study by [59] is another example of employing molecularly imprinted solid-phase extraction combined with gas chromatography. The authors designed MIPs to detect organochlorine fungicides in ginseng samples. This study involved four organochlorine fungicides (pentachloronitrobenzene, pentachloroaniline, methylpentachlorophenyl sulfide, and hexachlorobenzene) in ginseng samples. The newly synthesized molecularly imprinted polymer with pentachloronitrobenzene as the template achieved a low LOD of 0.001 mg kg^−1^ and LOQ of 0.002 mg kg^−1^. The method showed good recovery rates ranging from 79.3% to 95.2% for pentachloroaniline, 83.5% to 91.7% for hexachlorobenzene, and 80.3% to 90.4% for methylpentachlorophenyl sulfide, allowing for direct determination of these fungicides in ginseng samples.

The article by [60] presents an approach using MIPs combined with GC to efficiently extract and quantify progesterone hormones in diverse environmental and biological samples. The MIP was synthesized using FeCl_3_-assisted chemical oxidation of pyrrole, and its affinity-based selectivity toward progesterone was thoroughly investigated. Gas chromatography with flame ionization detector analysis was employed for precise determination without a derivatization step. The synthesized MIP exhibited high selectivity for progesterone over similar compounds, and the optimized parameters resulted in a low LOD of 0.625 ng mL^−1^ and LOQ of 1.875 ng mL^−1^. The method successfully monitored progesterone in environmental and biological samples, including tap water, hospital wastewater, urine, and blood serum. The recoveries for these samples were satisfactory, demonstrating the MIP’s suitability for extracting trace amounts of progesterone from complex matrices.

The research by [61] demonstrated the possibility of studying pesticides using GC and exploiting MIPs’ properties. A method for simultaneous separation and determination of four chloroacetamide herbicides (alachlor, acetochlor, pretilachlor, and metolachlor) in soil was proposed. The MIPs were synthesized via precipitation polymerization using butachlor as the dummy template, resulting in highly selective extraction and enrichment of the herbicides. The method demonstrated good linearity, with the LODs ranging from 1.0 × 10^−12^ to 5 × 10^−11^ g and LOQs from 0.0005 to 0.025 mg kg^−1^ for the four analytes.

GC-MS combines the separation capabilities of GC with the sensitivity and identification capabilities of MS. It is commonly employed for analyzing volatile and semi-volatile compounds. In MIP research, GC-MS is utilized for evaluating the efficiency of MIPs in the extraction and analysis of various target compounds. MIPs could also be used as the selective sorbents that are capable of identifying the preconcentration selected analyte, thus lowering the limit of detection and quantification of the analytical technique. The combination of GC and MS enhances the confidence in analyte identification and allows for the determination of trace amounts of analytes [62,63].

The work by [64] demonstrated one example of utilizing the GC-MS technique combined with MISPE. In this study, MIPs designed with *N*-nitrosodiphenylamine as the template were synthesized via precipitation polymerization. A method for determining *N*-nitrosodiphenylamine in water samples was developed using MIPs solid-phase extraction coupled with GC-MS detection. Under optimized conditions, the method showed average recoveries above 94% for the analyte spiked in ultrapure water at different concentrations, with an LOD of 0.8 ng L^−1^ and an LOQ of 2.4 ng L^−1^. The MISPE method demonstrated high selectivity and satisfactory recoveries of *N*-nitrosodiphenylamine in real water samples.

In another study presented by Shahhoseini [65], a method for analyzing 16 U.S. Environmental Protection Agency’s priority polycyclic aromatic hydrocarbons (PAHs) in water was demonstrated. The method involves using a tailor-made porous polymeric film for extraction and subsequent analysis via gas chromatography with atmospheric pressure chemical ionization–tandem mass spectrometry. The films offer good stability, and critical factors affecting extraction efficiency were optimized. The method demonstrates excellent analytical performance with low detection limits (LODs: 1–100 pg mL^−1^) and good reproducibility, making it suitable for high throughput environmental analysis of real water samples without the need for a standard addition. Moreover, the linear dynamic ranges cover a broad concentration range (1–50,000 pg mL^−1^), making it highly suitable for compliance with water quality regulations and environmental monitoring.

The following publication is an example of utilizing a dummy molecularly imprinted polymer (DMIP) strategy in SPE to recognize specific analytes [66]. This work focuses on the preparation DMIP technique to recognize the low-mass polybrominated diphenyl ethers PBDE-47 and PBDE-99. Four different DMIPs were synthesized using various functional monomers and porogen agents. The DMIPs demonstrated good sorption capacities, with imprinting factors (IFs) ranging from 1.1 to 4.0, and the recovery values of PBDE-47 and PBDE-99 were between 43% and 92%. GC-MS was employed to analyze PBDEs, and the LOD values were determined to be 0.0302 ng for PBDE-47 and 0.0315 ng for PBDE-99.

Another example of implementing the GC-MS method in food technology is found in the paper authored by Xu [67]. MMIPs were successfully synthesized as sorbents for selectively extracting hexamethylenetetramine from milk samples. The MMIPs were characterized, and their adsorption isotherms and kinetics were studied using GC-MS/MS. The optimized method using gas chromatography coupled with GC-MS/MS showed good linearity in the 1.0–50.0 μg L^−1^ range, with an LOD of 0.3 μg kg^−1^ and an LOQ of 1.0 μg kg^−1^. The recovery of hexamethylenetetramine from milk samples was between 88.7% and 111.4%.

### 2.3. Capillary Electrophoresis

Capillary electrophoresis (CE) is an analytical technique that separates and analyzes charged molecules based on the molecule’s charge, mass, and atomic radius. The particle’s migration speed is directly proportional to the strength of the applied electric field; higher field strength leads to faster mobility. Neutral species remain unaffected, as only charged ions respond to the electric field’s force. CE is a valuable tool for determining the composition and quantity of various compounds in a sample. The method involves the migration of analytes through a capillary tube filled with an electrolyte solution driven by an applied electric field. Capillary electrophoresis is an analytical method suitable for diverse applications due to its rapidity and minimal consumption of solvents and reagents. The drawback of CE is its sometimes higher LOD compared with other column techniques. However, various approaches involving sample preparation have been explored to overcome this limitation and improve efficiency. These techniques aim to lower the method’s LOD by pre-concentrating the analyte and removing interferences from the matrix. MIPs can be applied in various configurations when used inside the capillary. They can be coated onto the inner capillary wall; another technique involves forming a cylindrical monolith MIP in the capillary’s center; they can also be placed packed at the start, similar to a pre-column system, or MIPs can be utilized as tiny particles in the background electrolyte [68,69,70,71].

In [72], an MIP with high selectivity for trichlorfon, which is a pesticide residue in vegetables, was designed and synthesized. The MIP was compatible with the biomimetic immunoassay–capillary electrophoresis method, significantly improving the sensitivity for trichlorfon detection. The optimized conditions yielded an LOD of 0.16 mg L^−1^ for trichlorfon. The method was successfully applied to determine trichlorfon in kidney bean and cucumber samples, with recoveries ranging from 78.8% to 103%.

MMIPs can also be integrated with the CE technique. An approach employing MMIP was utilized to selectively extract atenolol from human plasma through magnetic solid-phase extraction [73]. Next, capillary electrophoresis was used, employing carboxymethyl-β-cyclodextrin as the chiral selector to separate atenolol enantiomers. The method exhibited an LOQ of 5.0 ng mL^−1^ for both enantiomers. The precision and accuracy of the method were well within regulatory requirements. Stability tests demonstrated the reliability of the method under specific storage conditions.

The following example of utilizing the CE method was reported by Bezdekova [74]. In this investigation, a method was invented for the selective isolation of phytoestrogens through noncovalent molecular imprinting. Through the use of genistein as the template and dopamine as the functional monomer, MIPs were successfully created in a microtitration well plate. The MIPs exhibited significantly higher binding affinity and selectivity toward genistein than nonimprinted polymers, as demonstrated by a calculated selectivity factor of 6.94. The proposed method was applied to isolate genistein from real bovine milk samples to evaluate its practical applicability. This isolation process was coupled with micellar electrokinetic capillary chromatography and UV-visible detection. The MIP-based technique displayed rapid adsorption of genistein, reaching saturation at 0.3 mg mL^−1^. The method exhibited a linear detection range of 2.2 to 27 µM, with a detection limit of 0.59 µM and a limit of quantification of 22 µM (R^2^ = 0.98).

CE combined with the MS system was proposed by Moreno-González [75]. The study presents a method for detecting patulin (a mycotoxin) in apple-based products at the µg kg^−1^ level. The method involves an in-line molecularly imprinted polymer solid-phase extraction micro-cartridge in capillary electrophoresis coupled with mass spectrometry. Validation parameters confirmed an LOQ of 1 µg kg^−1^ and a linear range of 1–100 µg kg^−1^ with high precision. The method successfully analyzed patulin in the presence of 5-hydroxymethylfurfural, which is a commonly interfering molecule.

The data from the discussed articles were collected and presented below (Table 1).

## 3. Mass Spectrometry Methods

### 3.1. Mass Spectrometry

Mass spectrometry (MS) is a technique that allows for the elaboration of the exact mass of targeted molecules. The general principle of the measurement is the conversion of the molecules into ions and then the investigation of their trajectories as a response to changeable fields—electric/magnetic or both. During the MS measurement, the key step is the transformation of the molecules into vapors. The vaporizing can be done using several methods. Therefore, both, the classical methods of ionization that are based on gas-phase encounters of the molecule to be ionized (by contact with an electron, photon, or electronically excited atoms, or even by chemical ionization), and so-called “soft” ionization methods that have been successfully used to produce ions from molecular species of increasing size and decreasing variability (e.g., plasma or laser desorption) can be applied [76]. In general, the MS technique has a long history of use; therefore, it shows broad applications in various fields. Due to J. J. Thomson’s pioneering work in laying the foundation of MS at the end of the 19th century, ion individuals’ generation has grown substantially over time. As a result, numerous variations of ion generation methods have been reported [77]. The forthcoming section elucidates some novel approaches for the determination of diverse contaminants, which is achieved via the amalgamation of the MS technique with MIP applications.

**Figure 3:**
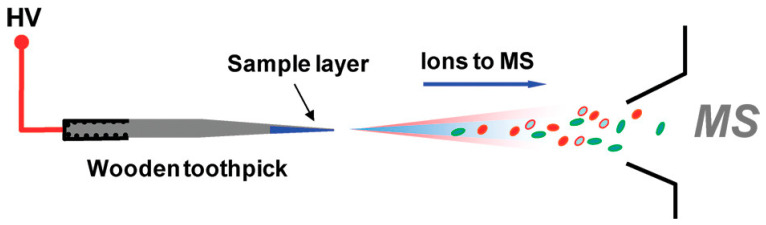
The general experimental setup of the wooden ESI-MS technique. Reproduced with permission from [78]. Copyright (2023), American Chemical Society.

Wooden tip ESI-MS is an ESI-based technique first reported in 2011 as a variation of a conventional method [78]. Whereas the ESI technique makes use of a capillary to deliver a sample for ionization under the high-voltage gas flow, wooden ESI-MS uses wooden toothpicks [79]. The conventional ESI-MS technique has found many applications for the analysis of various samples, and thus, has become one of the most frequently used methods in MS [80]. However, it has several limitations: (1) easily clogged capillary, (2) inconvenient loading of samples into the capillary, (3) time-consuming and labor intensity of the pretreatment for complex samples analysis, and (4) impossibility of the direct analysis of raw samples (e.g., biological tissue) [79]. On the other hand, the reported wooden ESI-MS showed many advantages from which low costs, relatively simple sampling, and compatibility with the various mass spectrometers must be mentioned. In addition, the use of wooden tips results in effective adhesion, thanks to its hydrophilic and porous nature, and the possibility of sampling from specific locations, thanks to its slim and hard properties. The proposed technique also overcomes the drawback of the impossibility of the direct analysis of raw samples via the use of standard ESI-MS. The wooden ESI-MS can be successfully used for raw biological samples and powder samples [78]. A general overview of the proposed wooden ESI-MS technique is presented in Figure 3.

One example of reported wooden tip ESI-MS is found in the article on trace malachite green and its metabolite products analysis from aquatic samples published by Huang et al. [81]. In this study, a molecularly imprinted polymer-coated wooden tip was proposed for the rapid and sensitive detection of targeted dye waste. The high toxicity of malachite green has significant implications for both human cells and the environment, underscoring the need for sensitive and rapid detection systems. In general, malachite green can induce liver tumor fabrication and is a potential threat to aquatic life [82,83]. The published report focused on using MIP-coated wooden tips for ESI-MS measurements. The developed unique, cost-effective, and portable probes for rapid and sensitive dye analysis allowed for its final detection from water and fish samples using real-time direct analysis. The proposed method shows the following advantages: simplicity, quantitativeness, reproducibility, and high throughput, showing the promising prospects of rapid and sensitive detection of malachite green and its metabolite in aquatic products [81].

Paper spray ionization mass spectrometry (PS-MS) is another example of the novel MS technique. In this variation, the samples are placed on a piece of triangle-shaped paper, usually made of cellulose of various thicknesses. The high DC voltage, reaching 3–5 kV, can be applied on the wet paper, which causes the release of charged analyte microdroplets, which can be subsequently transported to the MS for detection [84]. The PS-MS is thus an ambient ionization technique in which a spray solvent is used to wet triangle-shaped cellulose paper [85]. PS-MS shows important advantages that expand the utility of the MS technique, like (1) the possibility of the direct analysis of drugs and their metabolites from biofluids, (2) contaminant detection, and (3) bacterial discrimination without any special sample pretreatments. However, PS-MS shows some limitations. From the drawbacks, the limitation is the analysis of complex samples [86]. The problem can also be the presence of ion suspension during the analysis, poor sensitivity (especially at the residual level), and narrow dynamic linear range [87]. The PS-MS efficiency shows the dependence of many external factors, such as the angle of the paper spray tip, the solvent and paper composition (which affect the ion formation and signal stability), and the distance between the tip and MS inlet [88].

Mendes et al. [89] proposed an MIP-coated paper substrate for PS-MS to detect chosen human urine metabolites that correlate with several pathological conditions, such as heart disease, stress, neurological disorders, AIDS, and even tumors. The chosen metabolites can generally be treated as biomarkers; thus, their concentrations should be monitored. The proposed systems allow for detecting and quantifying dopamine, sarcosine, and butyric acid without any derivatization or complex sample pretreatment. As the literature data say, dopamine deficiency is associated with neuropsychiatric disorders (Parkinson’s and Alzheimer’s) and depression [90]. Furthermore, sarcosine is considered a prostate cancer biomarker [91]. On the other hand, the urinary levels of butyric acid may indicate bacterial confection in AIDS patients [92]. The obtained results from the three metabolites analysis showed suitable sensitivity, precision, accuracy, and recovery of the developed method [89].

**Figure 4:**
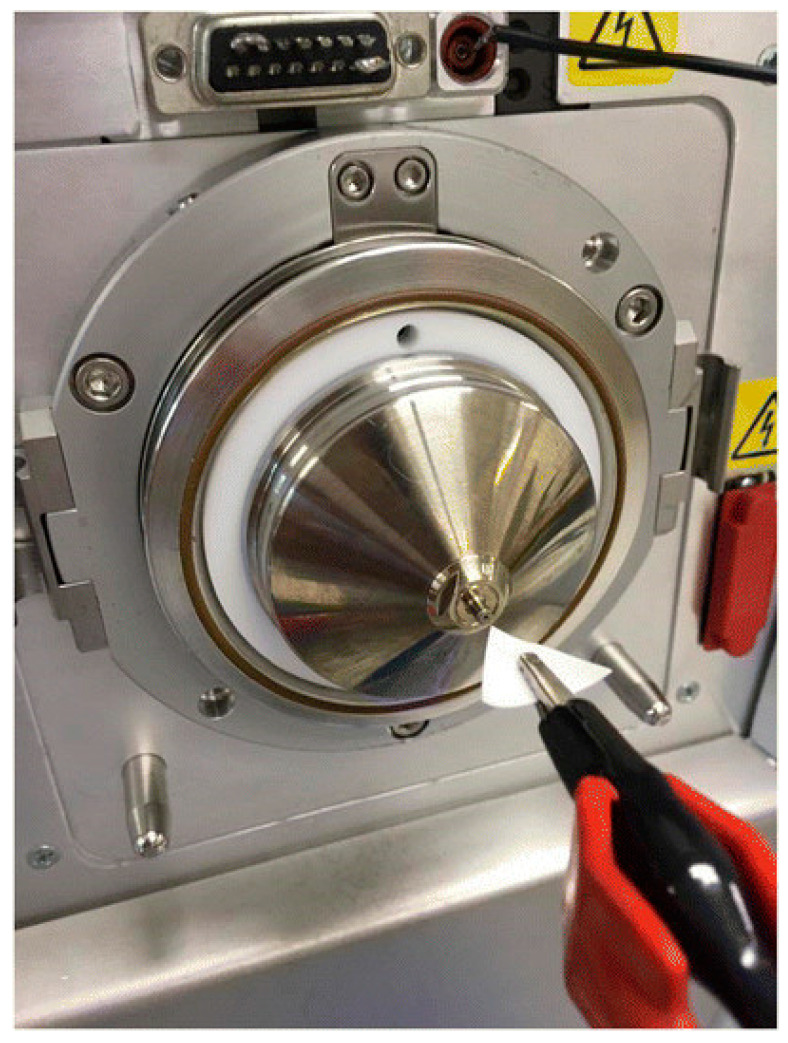
The general experimental setup of the PS-MS technique. Reproduced with permission from [93]. Copyright (2023), American Chemical Society.

Another important PS-MS application was reported by Tavares et al. [93]. In this study, an MIP-based cellulose membrane was developed to analyze cocaine in oral fluid samples directly. The report is an answer to the law changes of the non-tolerance to several psychoactive drugs in the blood systems of drivers operating a vehicle. Since the oral fluid is the ideal matrix to perform the cocaine presence assays, the report developed an adequate MIP-based system for the analysis. Among the advantages, oral fluid analysis provides rapid information [94], and its ease of collection makes it a non-invasive method [95]. The proposed PS-MS technique in this study creates new possibilities for paper spray ionization. Additionally, it confirmed the selectivity and specificity, simplicity, low-cost, low solvent consumption, speed, and efficiency of the proposed analysis. The apparatus used during the PS-MS measurements is presented in Figure 4 [93].

The investigation of the determination of phytotoxic substances, like diuron and 2,4-dichlorophenoxyacetic acid (2,4-D), in commercial food was proposed by Pereira et al. [96]. Detecting the mentioned agrochemical substances involved the utilization of apple, banana, and grape methanol extracts. The investigation relied on MIP-based cellulose membranes, where template molecules were incorporated into the synthesis process. During the MIPs synthesis, which was performed directly on a cellulose membrane, monuron and 2,4,5-trichlorophenoxyacetic acid (2,4,5-T) were used as templates for diuron and 2,4-D, respectively. The results showed that the diuron contents were only found in three bananas, reaching 4.0, 6.5, and 9.9 μg L^−1^. The proposed PS-MS technique with the combination of MIP-based membranes was straightforward, fast to carry out, and provided satisfactory results for analyses of targeted agrochemicals in apple, banana, and grape samples. Furthermore, the collected data were compared with non-imprinted polymers (NIPs), confirming the distinctive property of MIPs for the selective recognition of targeted analytes [96].

The expanded list of MIP-based MS sensors is presented in Table 2.

### 3.2. Ambient Ionization Mass Spectrometry

Ambient ionization mass spectrometry (ambient MS) consists of various techniques that allow for ion generation at atmospheric pressure. Moreover, the key advantages of these techniques include rapid, real-time, direct, and high-throughput analyses without any or minimal sample pretreatment. In addition, the samples can be measured directly from their surfaces or matrices. The targeted analytes are generally desorbed via laser desorption, thermal desorption, or impact by ions or charged droplets. The other important fact is that ambient MS is considered an ideal tool for surface composition or biological solids investigations, as the sampling process is noninvasive [99]. Whereas more than 30 ambient MS techniques are described in the literature, this review discusses only some interesting reports published in the past seven years. From the ambient MS family, the plasma-based techniques seem to be unique. In particular, direct analysis in real time (DART), flowing atmospheric pressure afterglow (FAPA), low-temperature plasma (LTP), and dielectric barrier discharge ionization (DBDI) should be mentioned as examples of strong interest. The mentioned techniques are based on the electrical discharge (direct current or radiofrequency) of a pair of electrodes in contact with flowing inert gas. Subsequently, the creation of a plasma species, namely, a stream of ionized molecules, radicals, excited state neutrals, and electrons, takes place. Finally, the obtained plasma species are directed toward the sample, which results in the desorption and ionization of targeted analytes. Compared with the ambient MS, the ambient plasma MS techniques show the following advantages: absence of solvents, simple instrumentation, rugged construction, and the generation of singly charged species that can be identifiable much more easily than multiple-charged ions generated by the use of other spray-based techniques [100]. Some of the examples of the use of ambient plasma MS are described as follows.

A direct current microwave argon plasma source for the use of MS was proposed by Guć et al. [101]. In this work, a microwave source was used to generate argon plasma for the thermal analyte’s ionization from the heated crucible with the temperature regulation from 100 to 250 °C. The aim of the study was the use of a proposed microwave argon plasma ionization source in ambient mass spectrometry for the direct analysis of pure compounds. The examined analytes were the templates from synthesized MIPs and commercial drugs in the form of tablets. The proposed MIPs were synthesized from commercially available compounds: methacrylic acid and acrylic acid as functional monomers; atrazine, pendimethalin, and quercetin as template molecules; and ethylene glycol dimethacrylate as a cross-linking agent. In the proposed method, analytes are thermally released from solid matrices without any sample pretreatment, followed by qualitative and quantitative analysis. Moreover, the analysis time has been significantly reduced without analyte loss, which is commonly observed in the classical sample isolation by the filtration or sludge spinning phases. The obtained results show that during the proposed analysis, the ionization occurs mainly through protonation and only with a small contribution of fragmentation and adduct formation. The proposed technique allows for the fragmentation analysis of low-molecular-weight compounds in complex matrices [101].

The other idea for quercetin determination by MS methods was proposed in a report also published by Guć et al. [102]. This study proposed the utilization of polyacrylamide-based MIPs, synthesized with the presence of the template molecule quercetin, for conducting ambient plasma ionization experiments. The proposed equipment was built with the following setup: a heated crucible, a FAPA plasma ion source, and an amaZon SL ion trap mass spectrometer. Using a heated crucible with programmable temperature allowed for the desorption of the analytes from the MIPs structure, which resulted in their direct introduction into the ion stream. Moreover, the experiments were done using MIPs and magnetic MIPs (mag-MIPs). In this report, the proposed FAPA-MS method was compared with that of the direct determination of quercetin in water solutions based on the use of ESI-MS. The final results show that the FAPA-MS application allows for the development of optimal analytical methods for the determination of trace analytes in diluted solutions, showing that this method is a modern tool enabling one-step, direct testing of organic substances contained in the solid phase [102].

**Figure 5:**
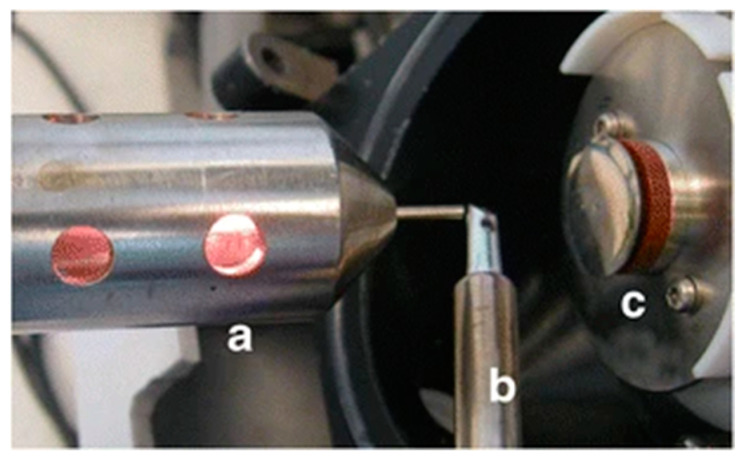
The general experimental setup of FAPA-MS technique: (a) FAPA ion source, (b) heating system, and (c) MS inlet. Reproduced with permission from [103]. Copyright (2023), Creative Commons CC BY license.

The first report on using MIPs as molecular scavengers for FAPA-MS was made by Cegłowski et al. [103]. In this study, three templates were examined (nicotine, propyphenazone, and methylparaben). The chosen templates were varied in terms of the origination and chemical formula to show the FAPA-MS technique’s feasibility. The physicochemical properties and adsorption characteristics of obtained MIPs were first examined during the research. Then, MIPs were transferred to the programmable heated crucible to induce the thermal desorption of samples. Obtained vapors were ionized and analyzed using FAPA-MS. This pioneering procedure allowed for bulk MIP analysis instead of MIP films that had been previously reported. Moreover, the use of reported bulk MIPs allows for the introduction of more analytes for analysis to improve the sensitivity of the proposed methodology. The photograph in Figure 5 presents the proposed pioneering equipment for performing FAPA-MS measurements. In general, the setup is made up of a FAPA ion source, a programmable heated mini-crucible, and the inlet to the MS. The proper arrangement of the whole setup’s elements determines the final results’ quality. Thus, the FAPA ion source should be positioned on the axis of the inlet of the MS with the tip located ca. 50 mm from the MS inlet. The programmable crucible should also be positioned ca. 10 mm below the ion steam to allow for temperature-controlled desorption. The obtained vapors were directly introduced into the plasma jet stream, which resulted in their ionization. The proposed technique analyzed both the positive and negative ions used during the studies. Whereas the nicotine and propylphenazone were investigated as positive ions, the methylparaben was analyzed in negative ion mode [103].

The collective findings from the application of the aforementioned FAPA-MS technique were as follows. The proposed method demonstrated swift MIP analysis, heightened LODs, and exceptional linearity for a broad spectrum of concentrations compared with alternative analytical techniques. Moreover, the calculated LODs indicate that the proposed analytical procedure allows for the detection of organic compounds, even at very low concentrations. As a result, the presented technique is also suitable for diagnostic purposes. One of the critical drawbacks is sample destruction, as the MIPs undergo complete thermal decomposition, which means they cannot be reused. However, considering that the MIP synthesis is relatively low-cost, this drawback can be considered minor.

Cegłowski et al. [8] also proposed another MIP-based platform for the selective determination and removal of potentially hazardous contaminants for environmental samples that can be subjected to FAPA-MS measurement. The proposed poly(2-oxazoline)-based MIPs were synthesized in the presence of a template molecule, namely, 2,4,5-trichlorophenoxyacetic acid (2,4,5-T). The idea was to prepare 4-(aminomethyl)-pyridine-functionalized poly(2-methoxycarbonylpropyl-2-oxazoline)s with various degrees of modification. The overall assumption was that the increasing amount of functionalization agent would result in the formation of a stronger interaction between the polymer chains and template molecule, and thus, the recognition property would be more effective. However, the results show that the highest values of the maximum adsorption capacity parameter were obtained for the lowest 4-AMP modification. Contrary to this result, the adsorption equilibrium coefficient increased with the functionalization degree, leading to high selectivity.

Other results for using the FAPA-MS technique were reported by Bogdanowicz et al. [104]. This study focused, first, on the synthesis of poly(methyl vinyl ether-alt-maleic acid)-based MIPs with 2,4-dichlorophenol as a template. Second, the obtained materials, differentiated by the amount of 4-AMP functionalization, were examined for their adsorption properties and then rapid quantification via the FAPA-MS method. Modified MIPs were synthesized with different degrees (10%, 20%, and 30%) of 4-AMP functionalization to investigate the influence of pyridine group content on the final adsorption properties. The experimental data indicate that maximum adsorption capacity was observed for the highest 4-AMP functionalization degree, as predicted. Similarly, MIPs with the highest 4-AMP content possessed the highest selectivity toward 2,4-dichlorophenol. Finally, all the obtained materials were used to quantify the analyte by their direct introduction into FAPA-MS. The LODs were improved significantly compared with the ones measured for the pure analyte solution (over two orders of magnitude). The proposed analytical technique was used to quantify 2,4-dichlorophenol in river water and wastewater samples. Good recovery results were obtained, showing that the method can be used to analyze complex real-life samples.

Examples of using the FAPA-MS technique for organic analyte determination by using MIP systems are presented in Table 3.

## 4. Electrochemical Methods

### 4.1. Voltammetric and Impedometric Methods

Electrochemical techniques have emerged as a cheap, easy-to-miniaturize, and simple alternative to traditional analytical validation methods for detection and quantification. While spectroscopic methods, electrophoresis, and chromatography are sensitive, they often suffer from limitations such as being time-consuming, complex to operate, and expensive. Although these conventional methods can offer good selectivity and low detection limits, they typically require complex pre-treatment steps and expensive instrumentation. In contrast, electrochemical techniques provide significant advantages, including convenience, cost effectiveness, and less time consumption. They do not necessitate sophisticated analytical facilities.

Electrochemical sensor techniques have emerged as an attractive solution, providing a cost-effective and easy-to-use platform for the sensitive, rapid, and selective determination of the desired analytes. These techniques can be integrated into reliable, compact, or miniaturized devices, enabling targeted applications in clinical and diagnostic settings. In particular, electrochemical sensors have a fast response time, making them valuable tools for diverse analytical needs while maintaining affordability [106,107,108].

Numerous voltammetric electrochemical techniques have been extensively documented in the scientific literature to analyze specific chemical compounds. The most frequently encountered methods are cyclic voltammetry, differential pulse voltammetry, and square wave voltammetry. These techniques involve the continuous manipulation of the applied potential to the solution via the electrode while simultaneously measuring the resulting current. Among these techniques, cyclic voltammetry (CV) stands out as the most widely utilized continuous wave approach to investigate redox reactions occurring in a solution. It serves as a valuable tool for acquiring critical data, such as oxidation and reduction mechanisms and the investigation of electron transfer kinetics. The CV method, which is commonly used for characterizing fabricated electrodes, is relatively less frequently employed in analytical performance measurements. Pulse techniques tend to offer higher sensitivity. Methods like differential pulse voltammetry (DPV) and square wave voltammetry (SWV) have shown exceptional usefulness in electroanalysis. The DPV technique stands out as one of the most widely employed voltammetric methods in the realm of MIP-based sensors. Its popularity is attributed to its simplicity, high sensitivity, and capability to diminish the noise originating from capacitive currents [109,110].

MIP-based voltammetric sensors have the remarkable ability to detect direct electroactive targets (conductive analytes) and indirect non-electroactive analytes. The template molecule can permeate the recognition sites within the imprinted membrane, allowing it to access the electrode surface and generate the corresponding electrical signal for the electroactive molecule. The quantitative analysis of template molecules can be achieved by observing the electrical signal’s magnitude. Additionally, for nonconductive molecules, their measurement can be accomplished indirectly using competitive measurements or by introducing specific electrochemical signal probes [111].

Sensors based on molecularly imprinted polymers were successfully characterized using the DPV [112,113,114,115,116,117,118,119,120] and SWV [121,122,123,124,125] techniques to quantify the analyte concentration. There are also examples where the cyclic voltammetry method was employed to assess the efficiency of the acquired sensor [118,126,127]. It is essential to establish a calibration curve to evaluate the performance of the obtained materials in the quantitative determination of analytes. This curve is obtained by measuring the current response in solutions with known analyte concentrations. For instance, in [112], the authors investigated solutions of the analyte using the DPV method over an analyte (tetrabromobisphenol-S) concentration range from 0.1 nM to 10 nM. They determined the maximum current intensity for each concentration. By constructing a linear function based on these data points, they obtained an R^2^ value of 0.9923. In the case of higher concentrations exceeding the linear range, applying an adsorption model, such as Langmuir, Freundlich, or Langmuir–Freundlich, is necessary. These models describe the adsorption behavior of the analyte on the electrode surface and can provide insights into the non-linear relationship between the analyte concentration and current response [128]. In many studies, optimization was performed to determine the influence of pH and incubation time on the obtained current values [114,121,124,126].

**Figure 6:**
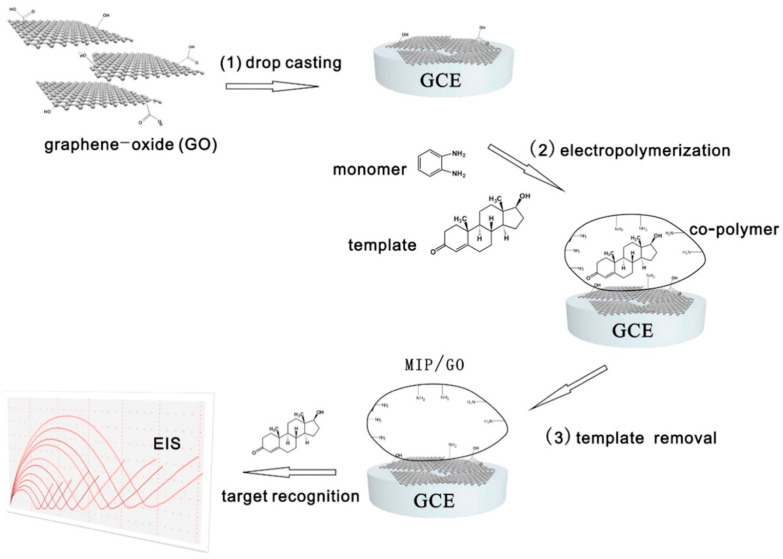
A procedure for preparation of testosterone-sensing impedometric transducer. Reprinted from [133]. Copyright (2023), with permission from Elsevier (or applicable society copyright owner).

Voltammetric methods commonly used to assess the performance of electrodes are often combined with electrochemical impedance spectroscopy (EIS) [112]. EIS is a technique that measures the impedance of an electrode–electrolyte interface as a function of frequency. By applying a small amplitude sinusoidal voltage perturbation and analyzing the resulting current response, valuable information about the electrochemical processes occurring at the electrode surface can be obtained. From the impedance spectroscopy, it is possible to determine the value of R_CT_ (charge transfer resistance), which changes with the analyte concentration in the solution. By measuring the impedance response at different analyte concentrations, variations in R_CT_ can be observed that correlate with the analyte concentration. For example, in the following study [129], a PBS solution containing [Fe(CN)_6_]^3−/4−^ ions was employed (Faradaic measurements). A fixed voltage of 0.22 V and a frequency range of 100 kHz to 100 mHz were established for the measurements. To construct the calibration curve, seven concentrations of the analyte (*N*-nitrosodimethylamine) were selected within the range of 10 µg L^−1^ to 230 µg L^−1^. Impedance spectroscopy measurements were performed on each solution, and the corresponding R_CT_ values were determined from the impedance spectra with the GCE as the working electrode. There are many other examples of using the EIS method to create an electrochemical sensor based on a molecularly imprinted polymer [130,131,132,133,134]. An exemplary illustration depicting the preparation of an EIS sensor based on graphene oxide and an MIP is presented below (Figure 6).

There are several methods for electrode fabrication or modification to enable the investigation of molecularly imprinted polymers as electrochemical sensors. Some commonly used techniques include using carbon paste electrodes mixed with fabricated polymers. In the work by Sarpong et al. [112], monomers (methacrylic acid and 4-aminothiophenol) were polymerized on the golden nanoparticles acting as the support and subsequently mixed with the carbon paste. Similarly, solid MIP particles were blended with carbon paste containing carbon nanotubes [125]. In another approach, monomers in the presence of template molecules can be polymerized directly on the electrode surface [126]. There are methods involving the modification of glassy carbon electrodes (GCEs) (for instance, by conducting electropolymerization on its surface) [113,114,116,121,123,133]. In the work by Liang [114], MIPs based on poly(methacrylic acid) and ethylene glycol dimethacrylate as the crosslinker were drop cast on a GCE covered by a graphene oxide layer. In [121], the authors modified a GCE using silver nanoparticles and then conducted electropolymerization of imidazole monomer in the presence of a template (17β-estradiol).

Another type of electrode that is mentioned in the scientific literature is the screen-printed electrode (SPEL). MIPs in [124,128] were either electrodeposited on or drop cast on the SPEL surface.

In the previously mentioned study [121], the authors achieved a highly efficient sensor capable of detecting 17β-estradiol. The produced sensor exhibited an exceptionally low detection limit with a value of 3.01 × 10^−7^ nM. The research utilized a modified glassy carbon electrode, incorporating silver nanoparticles and graphene oxide on its surface. The composite’s polymer component comprised polyimidazole, which was synthesized using electropolymerization. After eluting the template, the 17β-estradiol analytes were quantified by adsorbing them onto the specifically generated cavities using the SWV technique within the probe marker. The material demonstrated remarkable attributes, such as high percentage recoveries, sensitivity, repeatability, and ease of fabrication.

Liu et al. [133] also designed a highly promising sensor. The transducer was created by preparing a molecularly imprinted polymer through a straightforward electropolymerization process of *o*-phenylenediamine on a graphene-oxide-modified GCE. The resulting sensor demonstrated outstanding selectivity, high sensitivity (with a limit of detection equal to 4 × 10^−7^ nM), and long-term stability. It is worth mentioning that the analytical electrochemical method employed in the study was the EIS method.

Interesting results were obtained in the study by Motia et al. [115]. The authors carried out the polymerization of acrylamide in the presence of the herbicide triclosan. The synthesized material was then applied to a golden SPEL. To investigate the limit of detection for triclosan using the developed sensor, both EIS and DPV methods were employed. The LOD values were found to be 1.62 × 10^−2^ and 7.94 × 10^−4^ nM, respectively.

The summary of all the discussed obtained sensors based on polymers with a molecular imprint is presented in the table below (Table 4).

### 4.2. Field Effect Sensors

In recent years, the utilization of field-effect transistors (FETs) in conjunction with molecularly imprinted polymers has emerged as a powerful approach for analyzing and quantifying various analytes. Combining FET technology with MIPs, known as MIP-based FET biosensors or bio-FETs, is a novel method of sensing for analytical applications.

In the context of FET biosensors incorporating MIPs, potentiometry plays a critical role in detecting specific analytes and characterizing their interactions with the MIP recognition elements. Potentiometry involves evaluating and quantifying the concentration of ions in a solution by measuring the change in potential, typically under flow or batch conditions. Operating with minimal current flow, a potentiometric sensor detects the potential difference between the working and reference electrodes. This method allows for precise ion concentration determination. This unique capability allows for the straightforward detection of ions and small biomolecules as long as they carry intrinsic molecular charges. The principle of detection by bio-FETs is founded on potentiometric detection at the gate, where specific binding to target biomolecules leads to charge changes. The direct recognition of electroactive biomolecules captured within MIPs by MIP-FET biosensors showed rapid and concentration-dependent responses, underscoring their significance in direct characterization [135,136,137].

In [138], the researchers reported label-free and selective histamine detection using an MIP-coated gate field-effect transistor. As a monomer, the researchers used a methacrylic acid derivative, namely, 2-(trifluoromethyl)acrylic acid. Upon the addition of an initiator, cross-linking agent, and template, the scientists prepared a modified gold electrode with an MIP layer capable of selectively capturing histamine. The MIP-coated Au electrode was integrated with the silicon-based n-channel junction-type FET gate, and a gate voltage was subsequently applied via the Ag/AgCl reference electrode. As a result of this configuration, the histamine-imprinted MIP-coated FET showcased a highly responsive electrical behavior toward histamine concentrations equal to or exceeding 100 μM. This enhanced sensitivity facilitated histamine’s accurate and selective detection, particularly in scenarios involving mast cells’ allergic response. The research group utilized a similar method in other studies to analyze dopamine [139], as well as sugars such as 3’-sialyllactose and 6’-sialyllactose [140], and glucose [141].

In another example [142], the authors synthesized an MIP based on triphenylamine rhodanine-3-acetic acid as the monomer and epitopes of collagenase metalloproteinase-1 as the templates. The resulting sensors, constructed of MIP films integrated with FET, demonstrated excellent performance in detecting these epitopes, even in complex media, like control serum. The lowest limit of detection achieved was 20 nM.

It is also worth mentioning that this technique is useful for detecting individual enantiomers. The successful integration of MIPs with FET has been employed for the determination of both D- and L-phenylalanine [143]. Carboxy-derivatized bis(bithiophene) was employed as the functional monomer in preparing these MIPs. Remarkably, both phenylalanine enantiomers showed a detection limit of 13 μM. The enantioselectivity factor was approximately 2.3 for both chemosensors, demonstrating the ability of the MIP-based FET platform to differentiate between the enantiomers with satisfactory sensitivity.

## 5. Colorimetric Methods

Colorimetry is a widely used analytical method that determines the concentration of a chemical compound in a solution by employing color reagents. Chemical colorimetry establishes a relationship between the color characteristics of a tested sample and the concentration of its colored component. Depending on the sample’s properties, various characteristics can be measured, indirectly enabling the determination of even colorless substances through reactions that generate colored products. The diverse range of colorimetric characteristics related to color aspects allows for selecting the most suitable characteristic, resulting in enhanced sensitivity in substance determination [144]. By combining this unique methodology with MIPs, molecularly imprinted colorimetric sensors (MICSs) can be synthesized. These specialized polymers offer rapid, selective, and visual qualitative and semi-quantitative analyses of samples without the need for additional equipment. Additionally, highly sensitive quantitative analyses can be conducted using precision instruments. On the other hand, the combination of molecular imprinting technology (MIT) with colorimetric sensors significantly impacts the final properties of the obtained MICSs. The synthesized sensors exhibit high sensitivity and selectivity, fast response times, simplicity in handling and operation, and recyclability. Moreover, their preparation is relatively low-cost, making MICSs a focus of attention in the scientific world. A quick comparison with other detection methods reveals that colorimetry impacts visual color changes, making it suitable for daily-life measurements. However, the colorimetry sensor encounters issues with other interferences, limiting its application to liquid sample determination only. Furthermore, the sample’s composition must be straightforward, and color rendering should be resistant to interferences. Nevertheless, the unique combination of the colorimetric sensor with MIT can remarkably address this drawback, allowing for the analysis of more complex samples with great potential for practical applications [145]. Since MICSs are widely used to determine various contaminants, some interesting reports are presented as follows, divided into four categories: food safety, environmental analysis, biological sample detection, and drug detection and medical treatment.

### 5.1. Food Safety

Food safety has attracted widespread attention from the scientific world, as it consists of many essential aspects, such as human growth, development, and health. Before entering the market, a food product goes through many processes (production, transportation, packaging), and the food contaminations that can affect human health and development must be strictly controlled. Thus, the detection methods for food safety in daily life must provide good selectivity, low cost, low LODs, high sensitivity, easy portability, handling, and, most importantly, minimal reliance on instrumentation. These values can be achieved via the use of MICSs [145].

In one of the studies, a novel MICS-surface-enhanced Raman spectroscopy (SERS)/colorimetric dual sensor was proposed by Feng et al. to measure chlorpyrifos (CPF) in apple juice [146]. A colorimetric analysis based on color changes of synthesized silver nanoparticles (AgNPs) by interacting with CPF was proposed, while SERS spectra were directly collected by illuminating the AgNPs with a Raman laser. Since CPF is one of the most widely used organophosphate pesticides that can effectively inhibit acetylcholinesterase and block the signals traveling between nerve cells, its effective detection is required. Given that CPF is a pesticide that is moderately toxic to humans and is considered a neurotoxin, it may disrupt the endocrine system [147]. In the literature, there is a report that relates human exposure to CPF and the incidence of lung cancer [148]. Considering its toxicity and enormous application in agriculture, the effective detection of CPF residues in agri-food products is highly demanding. The proposed sensor allows for both the rapid, easy-to-operate screening and semi-quantification of CPF in apple juice (concentrations > 5 mg L^−1^) via naked-eye colorimetric methods and quantitative analysis (0.1–10 mg L^−1^) using a UV-Vis spectrometer. Moreover, the determination of CPF can be achieved within 25 min, including sample pretreatment [146].

A similar method for atrazine determination was proposed by Zhao et al. [149]. In this study, a novel dual-chemosensor was proposed as a method for the sensitive determination of atrazine in apple juice. By coupling MIP particles with instrument-free detection (gold nanoparticles (AuNPs)-based colorimetric assay) and an instrument-based quantification (surface-enhanced Raman spectroscopy (SERS)), an impressive LOD was achieved. The minimum registered LOD was 0.0012 mg L^−1^, which can meet the Health Canada requirements for the detection of atrazine in different foods within 25 min [149]. The widespread use of atrazine and its harmful impact on the hormonal systems of humans and animals necessitates strict control over the concentration of this artificial herbicide [150].

An interesting imprinted photosensor for phosphorus pesticide residue detection on the surface of vegetables and fruits was proposed by Huang et al. [151]. The long-range ordered macroporous inverse opal hydrogel particles were reported to obtain the sensor’s specific selectivity to methanephosphonic acid (MPA). This study introduced a novel approach by combining MIP colorimetric sensors with the hydrogen affinity process, resulting in a highly sensitive colorimetric sensor. The main objective was to develop a sensor capable of naked-eye detection of phosphorus pesticides, a promising application in food testing. An additional benefit of this sensor is its ability to efficiently detect the presence of MPA on the food surface, potentially reducing food poisoning accidents. In the experimental phase, the proposed MICSs were placed at the tip of a pipette. Subsequently, by utilizing capillary action, liquid from the food surface was drawn into the MIPCs, causing a noticeable color change that could be easily observed with the naked eye. The detection sensitivity and concentration range can be fine-tuned by adjusting the size of the hydrogel particles, and the detection limit can reach as low as 1.0 × 10^−6^ mol L^−1^. Additionally, the proposed analysis is easy to conduct and can be completed within 5 min using visual observation, eliminating the need for specific instrumentation [151].

In addition to the described examples, in recent years, MICSs were reported to be used as a part of the colorimetric determination of vanillin [152], caffeine [153], egg yolk antibodies [154], glyphosate [155], and ethyl-*o*-aminobenzoate [156].

In Table 5, some examples of the use of MIPs-based colorimetric sensors for quantifying analytes for food safety are collected.

### 5.2. Environmental Analysis

Due to continuous economic development, the presence of toxic and harmful substances in the environment is increasing. It has been established that certain commonly used substances pose risks to both human and animal survival. Consequently, strict control measures are necessary to manage pollutants introduced into the environment and safeguard the overall living environment. The utilization of MICSs (molecularly imprinted colorimetric sensors) in this industry enables on-site sample analysis, offering rapid results. Additionally, polymer platforms are relatively cost-effective and mitigate significant drawbacks, such as fluctuations, time consumption, and the need for extensive testing personnel and equipment [145].

**Figure 7:**
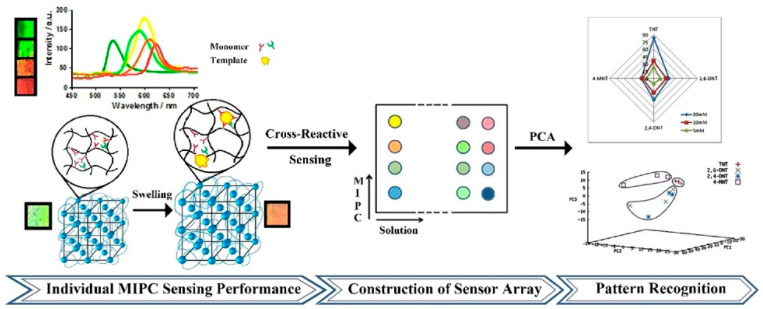
The overview of the proposed cross-responsive MICS-based colorimetric sensor for nitroaromatics detection. Reprinted from [157]. Copyright (2023), with permission from Elsevier (or applicable society copyright owner).

A novel MICS-based colorimetric sensor for nitroaromatics detection was proposed by Lu et al. [157]. In the proposed research, selective visual detection of 2,4,6-trinitrotoluene (TNT), 2,6-dinitrotoluene (2,6-DNT), 2,4-dinitrotoluene (2,4-DNT), and 4-nitrotoluene (4-MNT) were proposed. By observing the color changes of individual sensors depending on the analyte concentration, the evaluation was done using a “radar” pattern. This idea provided a comprehensive sensing result in a qualitative and semi-quantitative manner and further improved the selectivity of the individual sensor. The study also reported that the proposed MICS materials could display different colors from green to red in response to 20 mM nitroaromatics with varying shifts: 84 nm (TNT), 46 nm (2,6-DNT), 54 nm (2,4-DNT), and 35 nm (4-MNT). Following the use of principal component analysis (PCA) and rational design, the sensor array illustrated the influence of the nitryl quantity and generated a separate response region of nitroaromatics for pattern recognition with a good value [157]. The overall principle of the proposed approach is presented in Figure 7.

**Figure 8:**
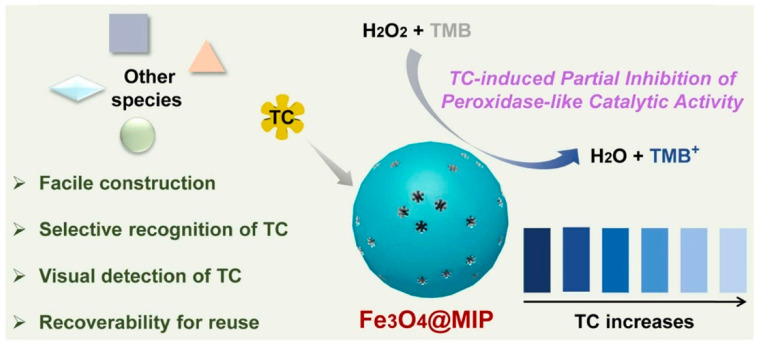
An overview of the proposed Fe_3_O_4_@MIP colorimetric sensor for selective tetracycline detection. Reprinted from [158]. Copyright (2023), with permission from Elsevier (or applicable society copyright owner).

Another interesting colorimetric sensor platform was proposed by Liu et al. [158], in which MIP sites were introduced onto the Fe_3_O_4_ nanozyme surface to obtain a colorimetric assay for tetracycline (TC) determination. It is a fact that TC is one of the most common antibiotics in animal husbandry, aquaculture, and human disease treatment. The universality of TC employed in animal husbandry, aquaculture, and human disease treatment, combined with the fact that over 60% of it is unabsorbed and directly excreted in the forms of urine and feces, caused it to pose a significant threat to the environment and living entities [159]. Therefore, it is of great significance to monitor TC to ensure food safety and ecosystem health. The overall mechanism for the colorimetric assay proposed by Liu et al. can be illustrated as follows. The proposed Fe_3_O_4_@MIP surfaces have cavities for substrate access to the active Fe_3_O_4_ core, which exhibits a good peroxidase-like catalytic ability to trigger the chromogenic oxidation of 3,3′,5,5′-tetrame-thylbenzidine (TMB). The crucial colorless’ TMB ability is that it can oxidate to show blue. When TC is added, it is specifically recognized and adsorbed by the MIP shell and partially blocks the accessible cavities. As a result, the catalyzed TMB chromogenic reaction is impeded [158]. The schematic diagram of the selective colorimetric detection of TC is shown in Figure 8.

According to the sensing principle, the colorimetric detection of TC with high selectivity against structural analogs was verified. The tests were also performed with the use of real-life samples, such as environmental water matrices. Moreover, due to the magnetic characteristics of the proposed sensors, they can be easily recovered and regenerated for recyclable use [158].

A magnetically responsive photonic crystal for bisphenol A sensing was proposed by Xu et al. [160]. The sensing mechanism was based on the color change with increased bisphenol A concentration. The structural color of the proposed sensor turned green to red, and the reflection wavelength shifted from 540 nm to 700 nm. The proposed method provided rapid analysis with good stability and reusability factors. Ten recognition/elution cycles were performed. Moreover, the sensor showed high sensitivity and selectivity parameters, rapid response, and easy operation; thus, it can be applied for the naked-eye detection of bisphenol A. The feasibility of detecting actual samples was also investigated [160].

Moreover, the MICSs were applied to other sensing systems to detect potentially hazardous contaminants in environmental samples. In recent years, MICSs were also applied for the colorimetric detection of tetrabromobisphenol A [161], pyrethroid pesticide [162], microcystin LR [163], and others.

The expanded list of MIP-based colorimetric sensors for environmental sample analysis is presented in Table 6.

### 5.3. Biological Samples Detection

Among the applications of MICSs, the determination of the composition and content of biological samples is considered crucial for human well-being. Analyzing biological samples provides essential information about health status, aiding in disease diagnosis, enabling timely detection and treatment, and thus, potentially saving lives. However, the complexity of the biological sample matrix poses a significant challenge in developing quantification methods that effectively detect low concentrations. A review of various methods reveals that many reported techniques are ineffective due to high costs, complexity, time-consuming processes, and reliance on large instruments and equipment. Therefore, there is a need to develop rapid, simple, portable, and cost-effective qualitative or quantitative methods [145]. The following examples illustrate how MICSs can fulfill these objectives.

A simple colorimetric ultrasensitive detector for viruses was proposed by Tang et al. [164]. As the accurate detection of viruses is considered a key factor in controlling and overcoming epidemics, the magnetic particles coated with carbon quantum dots were proposed as fluorescent sensors for colorimetric virus determination. The MICSs were introduced as a coating layer to enhance the specific recognition of the target virus. The overall mechanism used during the research is presented as follows. When the MIPs selectively recognized the targeted virus, the quenching of fluorescence of the carbon quantum dots was observed. As a result, the targeted enterovirus 71 with ultrasensitivity was detected. Moreover, the proposed method allowed for visual detection with the naked eye without any instruments and with a detection time of only 20 min. The proposed fluorescence-colorimetric dual-mode virus MIP-based sensor can be treated as unique equipment for epidemic prevention and rapid clinical diagnosis [164].

In one of the studies, a hydrogel colorimetric sensor was proposed to detect L-kynurenine in human serum without sample pretreatment. Rizvi et al. [57] proposed the selective binding of this metabolite as a solution to monitoring potential markers of immune suppressant disorder or even cancer [165]. This study confirmed that the selective binding of L-kynurenine resulted in shrinkage of the hydrogel volume and, respectively, a decrease in the particle spacing. It was found that this parameter can be monitored with the Debye diffraction ring diameter using a laser pointer. Hence, the proposed sensor demonstrated a visible red-to-green shift upon binding to L-kynurenine within 2 min. As a result, the reported colorimetric sensor showed highly specific and sensitive properties and allowed for rapid and convenient L-kynurenine detection [166].

In addition to the above examples, MICSs can be successively used for the rapid and selective determination of glycoprotein [167], thrombin [168], gonadotropin [169], horseradish peroxidase [170], glucose [171], and others.

An expanded list of MIP-based colorimetric sensors for biological sample detection is presented in Table 7.

### 5.4. Drug Detection and Medical Treatment

Typically, drugs are employed for disease treatment. However, as drug treatment becomes more widespread, their metabolites or undigested forms can persist in the environment, leading to environmental pollution. Consequently, a drug-contaminated environment may pose risks to human health. To address this issue, selective sensors were proposed for detecting drugs in various matrices, including human blood, urine, the environment, and food [145].

With the increase in antibiotic use over the years, there is a need to create special selective sensors for their detection. As the consumption and use of antibiotics have increased by both humans and the cattle industry [64], the concentrations of antibiotics in the environment have increased accordingly. One of the most significant risks is that the bacteria exposed consistently to these compounds become resistant to their destructive effects. Moreover, the transfer of antibiotics in animal-related byproducts, e.g., milk, has also been spotted [172]. For these reasons, Lowdon et al. [173] proposed a colorimetric MIP-based sensor for the selective determination of amoxicillin from environmental water samples. In this study, an MIP-based dye displacement assay for colorimetric detection in aqueous medium was proposed. The overall mechanism of the colorimetric assay was based on the mordant orange dye displaceability determined by the dose response. The research also provided selectivity confirmation, as the test was performed with the other compounds from the aminopenicillin class (ampicillin and cloxacillin). The findings from this research have the potential to be used in both a quantitative and semi-quantitative manner, depending on whether the analytical apparatus is on hand [173].

In the previous report, Lowdon et al. [174] proposed a platform for substrate displacement colorimetry. The study focused on detecting the narcotic compound 2-methoxiphenidine (2-MXP). The research studied the affinity of the MIP for six common dyes (malachite green, crystal violet, methyl orange, basic blue, pararosaline, and phenol red) expressed as the binding factor. The general result indicated that the relationship between this factor and the imprinting factor can be used to predict the efficacy of the displacement assay. The displacement test was performed as follows. Dye-loaded MIPs were incubated with the target analyte (2-MXP), two adulterants, and two legal pharmacological compounds. As the MIPs possessed a higher affinity toward amoxicillin, the dye particle was displaced out of the cavities of the receptor leading to a color change in the filtrate that could be observed with the naked eye. The proposed substrate displacement colorimetry assay was robust, fast, low-cost, and highly selective. The study confirmed that the proposed methodology could be successfully used as a pre-screening tool to identify narcotic substances in unidentified powders [174].

Given the demands of modernity, a smartphone-based colorimetric detection assay was proposed by Karim et al. [175]. The detection mechanism was based on the synthesis of two MIPs, which differ by the templates. Whereas one of the templates was acetazolamide, whose determination was the scope of the research, the second one was its analog sulfamethoxazole because of its ability to transform into a colored product. Finally, the smartphone was employed for taking images and analyzing color intensities via RGB color application [175].

A list of recently reported MIP-based colorimetric sensors for drug detection and medical treatment is presented in Table 8.

## 6. Plasmon Resonance Methods

**Figure 9:**
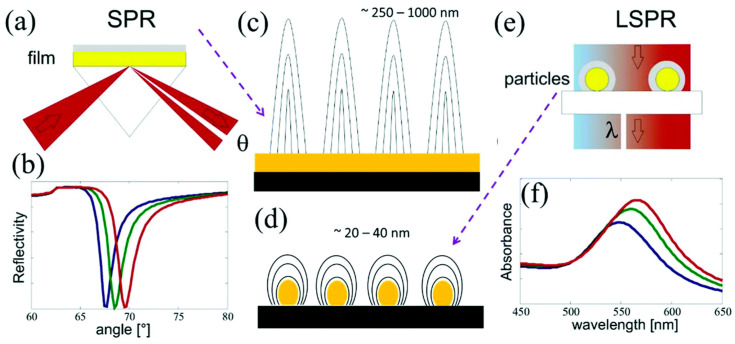
Overviews of SPR (**a**–**c**) and LSPR (**d**–**f**) mechanisms. Reproduced with permission from [180], open access Creative Common CC licensed 3.0, Royal Chemical Society (RSC)).

Surface plasmon resonance (SPR) is a very sensitive technique that allows for the determination of small refractive index changes at the interface between an analyte (dielectric medium) and the surface (metallic layer) [176]. This technique is widely used in many biosensing and chemical sensing applications. Generally, the real-life examination of biological samples is based on transport through a carrier or buffer fluid that serves as a microfluid system. As a basic rule, the SPR sensors consist of a transducing media bounded to the metallic layer. The subsequent reaction of this media with the targeted molecules from the analyte causes changes within the refractive index at the outer interface. The resulting change can be detected via a suitable optical interrogation [177]. The factor of vital importance is the distance between the dielectric medium and the metallic layer. Thus, the sensitivity of the plasmonic phenomenon decreases with the distance increase, as the effective interaction length should not be longer than a few hundred nanometers [178]. In general, the surface plasmons systems are free electron oscillations of the surface, and the analytes can be classified into the two following categories: (1) propagating surface plasmons and (2) localized surface plasmons [179]. The overall mechanisms of both techniques are presented in Figure 9.

At first, the basic rule is that SPR sensors use the evanescent field of surface plasmons propagating through a metallic surface. It allows for detecting the sample’s dielectric variations localized around 100 nm from the plasmonic material. As a result, the interaction of the evanescent field with the analyte causes a shift in the transmitted light’s wavelength or a change in the light intensity. Both observed results should be proportional to the changes in the sample’s refractive index, which allows for detecting the target analyte [181,182,183].

On the other hand, localized surface plasmon resonance (LSPR) is a phenomenon of excitation on nanoparticles, which can subsequently induce a strong electromagnetic field, leading directly to extensive applications in surface enhancement. This phenomenon is based on the nanoparticles’ size, which has to be similar to the light’s wavelength. Thus the particle’s free electrons contribute to the coherent oscillation [182].

Among others, two significant LSPR effects need to be mentioned: (1) the electric field close to the nanoparticle’s surface is considerably enhanced and (2) the nanoparticle’s optical transmission spectrum shows a maximum at the plasmon resonant frequency in the region of the UV-Vis (depending on the refractive index of the surrounding medium) [184]. The general principle of LSPR sensing is based on the plasmon resonance spectral shift. This shift can be determined via a change in the dielectric properties of the environment surrounding the metal nanoparticles [185].

The MIP’s ability to selectively recognize targeted molecules at trace levels without the necessity of sample pretreatment shaped the frequency of the MIP applications in many fields. Therefore, the combination of them with the peculiar features of fiber-optic-based sensors, such as allowing remote or online monitoring and the possibility of obtaining miniaturized devices suitable for point-of-care measurements, has led to huge interest in applications in several fields, like the environment [186,187], agriculture and food industries [188], energy, pharmaceutics [189], and medicine [190,191], in recent years. Because from the optical MIPs-based sensors, the SPR and LSPR are thought to be the most promising techniques [192], some innovative examples of their use from the past 7 years are presented below.

### 6.1. Surface Plasmonic Resonance (SPR)

As a response to the consistently increasing problems with antibiotics pollution in the ecosystem, a novel sensing MIPs-based platform was reported by Ayankojo et al. [189] for the detection and subsequent investigation of amoxicillin using SPR sensors. A hybrid organic–inorganic MIP chemosensor was synthesized for the selective recognition of amoxicillin. The hybrid MIPs film was synthesized using a sol-gel from a methacrylamide (MAAM) monomer, which was used as an organic monomer; tetramethylsilane (TEOS), which was treated as an inorganic precursor; and vinylmethoxysilane (VTMOS), which is an inorganic coupling agent. Thanks to the sol-gel methodology, the obtained flexibility in the MIPs design promotes the development of a highly porous structure, and thus, increases the concentration of functional groups in the surface area. It directly results in better analytical sensitivity and selectivity [193]. Additionally, the use of hybrid, silica-containing MIP films is considered inert to the environment due to silica’s nature. Also, the optical transparency of a sol-gel hybrid silica MIP film caused it to be suitable for the SPR technique. The proposed MIP sensor was assessed regarding the selectivity parameter and limit of detection (LOD) to confirm its unique feasibility for detecting amoxicillin in environmental samples (e.g., tap water). The proposed chemosensing MIPs-based materials demonstrated about 16 times higher binding amoxicillin capacity than the corresponding non-imprinted reference materials. The obtained LOD was 73 pM. Reported SPR sensors can discriminate amoxicillin among structurally similar molecules in a buffer and tap water. The additional benefit is the stability of the proposed sensors, where the proposed materials can be stored at room temperature for up to 6 months [189].

Other chemical compounds that should be strictly monitored in environmental water are perfluorinated alkylated substances (PFAs), which are highly dangerous for ecosystems, biodiversity, and human health. According to the EU directive 2013/39/UW, within the next 20 years, PFA compounds should be eliminated; thus, the investigation for their selective removal from the ground and surface water is essential. The examples of well-studied PFAs are perfluorooctanoate (PFOA) and perfluorooctanesulfonate (PFOS), for whom selective removal was the aim of the studies presented by Cennamo et al. [186]. In this report, a novel low-cost MIPs-based sensing platform was synthesized on a plastic optical fibers (POFs) matrix. For the MIPs synthesis, two functional monomers were used, namely, (vinylbenzyl)trimethylammonium chloride and 1H,1H,2H, 2H-perfluoroalkyl acrylate, in the presence of a template, namely, ammonium perfluorooctanoate. As a cross-linking agent, ethylene glycol dimethacrylate was used. The applied POFs show many advantages, from which, low cost and simplicity in manufacturing, easy handling and installation procedures, and large fiber diameter (a millimeter or more) should be mentioned as the most valuable [194,195]. An additional MIP advantage, namely, the possibility of their direct deposition on a flat gold surface without modifying the surface, which is valuable for bio-receptors, caused the use of this system in this report [196]. A novel MIP that is able to bind perfluorinated compounds, combined with an SPR optical fiber platform, was proposed. The results showed selectivity and the ability to sense targeted compounds at very low concentrations, with an LOD down to 0.13–0.15 ppb [186].

Since using sensor platforms based on optical fibers for the effective, online detection of perfluoroalkyl and polyfluoroalkyl substances is relatively quick and easy, it has become more attractive in recent years. Compared with other well-known techniques that were used to detect the PFAs, such as high-performance liquid chromatography–mass spectrometry (HPLC–MS) and gas chromatography–mass spectrometry (GC–MS) [197,198], fluorescence-based sensors [199], or electrochemical impedance spectroscopy (EIS) [200], only the use of SPR overcomes common existing disadvantages. These drawbacks are (1) time consumption, (2) the requirement of laborious preparation steps, and (3) employing a large number of solvents [201]. In addition, there is high interest in the selective detection of PFAa results from the properties of these compounds that directly affect human health and the ecosystem. First, PFAs contain carbon–fluorine bonds, which are the strongest chemical bonds in organic chemistry. Due to a lack of natural decomposition in the environment, they have been classified as persistent organic pollutants (POPs). Second, PFAs are widely used in common daily-life applications. They can be found in drinking and surface waters, oils, food packaging, cookware, or human food sources [202]. The result is that they can accumulate in human organs through this continuous exposure, leading to severe health risks even at low concentrations. Furthermore, the present PFAs in the environment can create dangerous derivatives, which can subsequently cause bioaccumulation, as they are resistant to various biological and chemical treatments [203]. In another report, two different POF platforms were proposed by Pitruzella et al. [201] The first proposed platform was a chemical chip obtained using POF with microholes filled with a specific MIP, and the second was an SPR sensor based on a D-shaped POF. The materials were synthesized according to Cennamo et al. [186], but contrary to this report, the obtained MIPs were deposited in three microholes in the proposed D-shaped POF’s core instead of the SPR-POF probe. The resulting binding changes within the MIP particles influenced the chemical chip’s refractive index, causing a variation in the plasmonic resonance. The idea of the location of the MIP particles in direct contact with the core instead of the gold surface of the SPR-POF probe resulted in better detection limit ranges, which were reported as 0.0063 nm/ppt [201].

In the current literature, some reports publish the detection of compounds that should be controlled in food or drinks. One of the great examples is an article reported by Pesavento et al. in which furfural in wine was detected from an SPR-optical fiber-MIP sensor. The report proposed a novel SPR platform based on a D-shaped POF, combined with a biomimetic MIP receptor to detect furfural in fermented beverages. The MIP particles were prepared from methacrylic acid as a monomer in the presence of a template, namely, 2-formaldehyde (2-FAL). As a cross-linker, divinylbenzene was used. The study’s main aim was to measure 2-FAL in real-life samples, such as wine. The results show a low detection limit of 0.004 mg L^−1^ [188].

### 6.2. Localized Surface Plasmonic Resonance (LSPR)

In one of the promising works reported by Culver et al. [204], a method for synthesizing poly(*N*-isopropylacrylamide-*co*-methacrylic acid) (PNM) hydrogels for binding a protein on the surface of silica gold nanoshells (AuNSs) was developed. Then, the ability of these materials as both molecular recognition and signal transduction agents was tested. The proposed materials were used to detect the changes in the concentration of two common chronic dry eye protein biomarkers: lysozyme and lactoferrin. The main aim of the research was to produce affordable LSPR biosensors for rapid and easy use for medical applications for point-of-care diagnostics [205]. The idea of conducting research was based on the changes in the shifts in the LSPR wavelength. After the targeted biomarkers’ binding, the proposed materials exhibited significant red wavelength shifts depending on the concentration. As a result, the observed LSPR wavelength shifts enabled the detection of the investigated biomarkers in human tears. The proposed LSPR-based biosensor can be used as an effective tool for chronic dry eye and associated conditions. Compared with the previously reported LSPR-based biosensors, the proposed material exhibited significant LSPR shifts reaching up to 50 nm upon analyte binding. These observed shifts resulted in high analyte adsorption capacity and drastic changes in the refractive index [204].

An interesting proposition of the use of sensor devices combining LSPR and MIPs was reported by Guerreiro et al. [206]. The research aimed to evaluate wine astringency at the molecular level. While polyphenols are thought to be responsible for the color, smell, and taste of most food products, the astringency is also strictly correlated with the presence of this type of compound [207]. Thus, the investigation was based on the interactions between the salivary proteins and the pentagalloyl glucose (PGG) and polyphenols present in wine samples. These interactions were used for the LSPR sensor calibration and then direct wine samples analysis and, as a result, astringency establishment. The salivary protein was imprinted into the methacrylic acid (MAA) polymer, cross-linked using the EGDMA, and placed on a gold nanodisc using a surface imprinting technique [206].

A combined list for both SPR and LSPR MIP-based sensors is presented in Table 9.

## 7. Surface-Enhanced Raman Spectroscopy

Surface-enhanced Raman spectroscopy (SERS) is an analytical technique based on the principle of enhancing the Raman signal of molecules adsorbed on or near certain metallic surfaces, such as silver or gold nanoparticles. The SERS effect combines two phenomena: electromagnetic enhancement and chemical enhancement.

Electromagnetic enhancement arises from the localized surface plasmon resonance (LSPR) of the metal nanoparticles. When molecules are in close proximity to the metallic surface, they experience an enhanced electromagnetic field. When a laser beam is used to excite the sample, the incident light interacts with this field, significantly increasing the intensity of the Raman scattering. Chemical enhancement, the other aspect of SERS, is due to chemical interactions between the analyte molecules and the metal surface. These interactions can alter the molecular polarizability and affect the Raman scattering cross-section of the molecules, further contributing to enhancing the Raman signal. The SERS effect heavily relies on the properties of the metal substrate, the distance between the analyte molecules and the metal surface, and the laser excitation wavelength used. SERS has gained widespread popularity due to its attributes, including high sensitivity, rapid response, and the ability to provide distinctive “fingerprint” recognition of molecules. The remarkable compatibility of SERS with water systems makes it particularly advantageous for analyzing aqueous samples. Additionally, the linear relationship observed between the spectral intensity and analytical concentration further enhances its appeal, enabling SERS to be effectively utilized for qualitative, semi-quantitative, and quantitative analyses [208,209,210].

The combination of SERS with MIPs offers several advantages for analytical purposes. SERS provides high sensitivity for detecting low concentrations of analytes. When integrated with SERS-active substrates, it allows for detecting even trace amounts of target molecules, enhancing the sensitivity. Moreover, the selectivity of MIPs, along with the Raman signal enhancement of SERS, enables the selective detection and identification of the target analyte in complex samples, reducing interference from other components. Conventional MIP-SERS sensors consist of two main elements: SERS substrates and MIP layers, where the MIP layers are applied onto the substrates. These sensors operate in two primary detection modes. The first mode is the label-free approach, which is suitable for analytes exhibiting strong Raman signals, such as molecules with significant chromophores. In this mode, analyte identification and quantification can be directly achieved based on their inherent signals. However, direct signal monitoring is not feasible for molecules with smaller cross-sections. To overcome this limitation, SERS probes with high sensitivity and distinctive Raman signatures play a critical role. These probes serve as a labeling mode in qualitative and quantitative detections, facilitating the identification and quantification of analytes with smaller cross-sections [211,212].

An exemplary work that involved implementing the MIP-SERS method in analytical chemistry was performed by Hua [213]. This study introduced a novel approach using MIPs and SERS for the rapid and sensitive detection of 2,4-dichlorophenoxyacetic acid (2,4-D) residue in milk. The method achieved a limit of detection of 0.006 ppm and a limit of quantification of 0.008 ppm for 2,4-D in skimmed milk. It offers a simple and efficient solution for detecting trace amounts of 2,4-D in food. The MIPs were synthesized through bulk polymerization and utilized as a solid-phase extraction sorbent to extract and enrich 2,4-D from milk samples selectively. Additionally, silver nanoparticles were synthesized to facilitate the collection of the SERS spectra of the extracted samples. The entire 2,4-D testing process for each milk sample required only 20 min, making this MIP-SERS method highly efficient and practical for detecting 2,4-D in agri-foods.

A novel idea was implemented in the following work [214]. A biosensor combining SERS with MIP technology was developed to detect a tumor biomarker, namely, carcinoembryonic antigen, quantitatively. The biosensor showed a low limit of detection (LOD) of 0.064 pg mL^−1^ and a broad detection range of 0.1 pg mL^−1^ to 10 μg mL^−1^. It demonstrated excellent performance in detecting CEA in real blood from cancer patients, holding promise for biomarker-based cancer screening.

An interesting approach was proposed by Zhang [215]. In this study, a method was developed to detect malachite green in fish muscles. The optimized MIP demonstrated significantly higher adsorption efficiency for malachite green compared with nonimprinted polymers. Gold-silver core–shell nanoparticles with a 74 nm diameter were used as the substrate to enhance the SERS signals. Remarkably, the MIP-SERS method achieved an impressive detection limit of 0.5–1 ng g^−1^ for malachite green in fish muscles, meeting the sensitivity requirements set by the European Food Safety Authority. This innovative approach provides a rapid, selective, and highly sensitive solution for detecting malachite green in aquatic products, ensuring food safety. Moreover, its potential extends to identifying other restricted hazards in various food items, making it a valuable tool in food safety assessment.

## 8. Fluorometric Methods

In general, fluorescence can be understood as the emission of light by an atom or molecule following the absorption of electromagnetic energy. To clarify, fluorescence occurs as the excited species transitions from its initial excited electronic singlet level to its ground electronic level. This technique offers numerous advantages that render it highly valuable for the examination of molecular processes. Among the various aspects to highlight, the persistence of the excited state for nanoseconds duration should be emphasized. While the phenomenon of fluorescence has been observed for an extended period, its recent surge in popularity can be attributed to the advancement of highly sophisticated fluorescent probe chemistries, the widespread commercial availability of these probes, and the development of innovative microscopy techniques [216]. In the age of scientific exploration aimed at sensor-based devices, which promise a multitude of advantageous attributes, including straightforward implementation, swift processing times, elevated sensitivity, and selectivity, fluorescence-based sensing emerges as a viable alternative. As this technique additionally shows a short response time, low cost, and simplification [217], it has been applied in various fields, such as biomedical diagnosis [218]. Through the distinctive fusion of the fluorescence technique with MIP materials, there exists the potential for substantial enhancement in selectivity. The subsequent section delves into the sensors based on MIPs that were reported for the application of the fluorescence technique [219].

In one of these studies, a novel metal-organic framework capped with an aptamer and MIPs was designed for malachite green detection by Duan et al. [220]. In this study, initially, a fluorescence-based sensor was fabricated and employed as a carrier for the conjugation with the malachite green aptamer. Subsequently, a template was introduced to create an MIP film layer through a copolymerization process. The resulting material was utilized for the precise quantification of malachite green through fluorescence emission. The concept behind this approach was to achieve synergistic binding of the template with both the aptamer and MIP, thus inducing a photo-induced electron transfer (PET) effect. Furthermore, the resulting fluorescence sensor was integrated into circular-patterned paper and assessed using a smartphone equipped with color recognition software to read the RGB values from the captured images. The proposed research showed that the introduction of aptamers into the MIP layer enhanced the interaction of the template molecule and the imprinted cavity due to its high affinity and specificity, and the synergistic binding effect of aptamer and MIP improved the interactions and the final intensity, and thus, the high sensitivity. Moreover, the unique combination of the paper substrate and smartphone readout provides fast, portable, and easy-to-operate detection [220].

An interesting fluoride-selective chemosensor was proposed by Quiñone et al. [221]. This study was the first report on the design of a polymeric optical fluoride sensor using the anion-imprinting technique, which is thought to improve the sensitivity and selectivity of the device. The idea was to synthesize the urea/thiourea-based anion sensors for fluoride detection, as the sensing of this ion has become a significant analytical goal due to the fact that this anion can be emitted into the environment by the chemical industry, medical processes, and the degradation of nerve agents used in terrorism [222]. Therefore, monitoring the levels of fluoride ions in victims, drinking water, and the surrounding environments adds significant value [223]. The experiments were conducted using four mixtures of polymerizable anion receptors, with each of them employing distinct fluorophores as signaling units. The results obtained from the primary fluoride ion detection were contrasted with those for other anions. The findings revealed that the fluorescent emission was 13 times more pronounced in the presence of the polymer and was exclusively quenched by fluoride ions, whereas there was virtually no optical response to the remaining anions [221].

Another sensitive fluorometric method for quercetin was proposed by Karrat et al. [224]. In this research, a novel magnetic MIP for selective extraction and determination of quercetin in plant samples was proposed. Given that quercetin possesses robust antioxidant, anticancer, antiallergic, anti-inflammatory, antiviral, and cardioprotective properties, it holds potential utility in the realms of both medicine and pharmaceuticals [225]. Nevertheless, extracting and isolating quercetin from plant extracts presents significant challenges. Consequently, the development of a selective, rapid, straightforward, and precise method for quercetin extraction and detection is greatly sought after and aligns with the objectives of the presented studies. The obtained magnetic MIP was combined with the spectrofluorometric method for the selective extraction and detection of quercetin in plant samples [224].

Exemplary work involving implementing a fluorescence test strip for the determination of tyramine was proposed by Chen et al. [226], which is important due to tyramine’s cytotoxicity [227]. The idea involved the creation of paper-based sensors incorporating MIPs for the detection of specific biogenic amines. The proposed sensor was built using the green fluorescence of upconversion nanoparticles (UCNPs) and the specific recognition property provided by MIPs. The MIP film was synthesized via in situ polymerization, utilizing tyramine as the template and methacrylic acid as the monomer, which was cross-linked with EGDMA. This amalgamation of materials offered several advantages, including minimal interference from matrix substances due to UCNPs, heightened selectivity due to MIPs, and user-friendly operation in a standard test strip format. Furthermore, the applicability of the proposed fluorescence paper-based sensor was demonstrated in the detection of tyramine within complex real-life samples (red wine, mature vinegar). The demonstrated portability, cost-effectiveness, and swift response of the proposed approach make it highly promising for the future [226].

A list of recently reported MIP-based fluorescence sensors for organic contaminants is presented in Table 10.

## 9. Challenges

MIPs combined with analytical methods have gained significant attention in recent years, offering a range of perspectives, challenges, and applications. MIPs hold the potential for selective preconcentration, which is a capability that enhances detection sensitivity and specificity within analytical techniques. However, attaining this selectivity can prove challenging, especially when confronted with structurally similar compounds. Furthermore, MIPs simplify complex sample matrices, streamlining the detection and quantification of specific analytes. Nevertheless, the real-world complexity of samples, which are often filled with interfering substances, can impact MIP performance [7,10,217].

Their versatility is a standout feature, with MIPs being tailorable to a broad spectrum of target molecules, affording versatility in various applications. However, their synthesis process can be complicated and time-intensive, necessitating optimization for each unique analyte. The current state of MIPs is one of active research and application across diverse fields, including pharmaceuticals, environmental monitoring, and food safety, with ongoing advancements in MIP design and integration. Persistent challenges involve mitigating matrix effects that can affect MIP performance, addressing the details of MIP synthesis for optimal outcomes, and overcoming limitations related to the primarily non-covalent interactions on which MIPs rely, particularly in complex sample matrices [7,231,232].

The synergy of MIPs with analytical methods holds vast potential for enhancing analyte detection and quantification. Nevertheless, the effective harnessing of this potential centers on overcoming challenges related to selectivity, synthesis refinement, and matrix effects. The future beckons with prospects such as innovations in point-of-care diagnostics, security applications, and biotechnology advancements, all of which are driven by the continuous evolution of MIP technology [233,234,235].

## 10. Conclusions

In summary, this comprehensive review highlights the versatility of MIP-based analytical procedures. It is substantiated by numerous significant studies conducted over the past seven years, employing diverse analytical techniques to achieve precise analyte determination. This review vividly illustrates the widespread utility of MIP sensors, showcasing their applicability across various analytical methods. The choice of an appropriate analytical methodology hinges on several parameters, including time, cost factors, detection methods, and inherent sample attributes. The methods currently employed exhibit a wide range of LOD values, which can differ significantly even within the confines of a single technique. Consequently, a general principle can be inferred, namely, that the LOD value is influenced by several factors, with the choice of the technique not being the sole determinant. The selected technique needs to be appropriately modified based on the intended application, necessitating corresponding adaptations in the detection method.

Analytical methods combined with MIPs inherit the same limitations and advantages as methods that do not utilize MIPs as the active material. The time associated with the actual analytical procedure is usually independent of the use of MIP. In the methods described in this study, the primary objective of using MIPs is the localized preconcentration of the target analyte to reduce the LOD of the particular analytical technique. The MISPE technique, which exemplifies the application of MIPs for this purpose, can be integrated with various analytical methods. For instance, incorporating MISPE into HPLC enables the selective accumulation of the analyte within a relatively small space (the surface of MIPs). Controlled elution subsequently leads to a significant increase in analyte concentration within a short time frame. In the case of techniques like SERS, enriching the eluate with the analyte and combining it with metallic nanoparticles allows for a larger coverage of these nanoparticles, resulting in enhanced Raman signal amplification. Another important consideration when comparing analytical methods that employ MIPs is the sample preparation time. For instance, in electrochemical methods, MIPs are an integral part of the electrode, often requiring thorough preparation, including the time needed to generate a suitable conductive layer containing MIPs. These methods often necessitate the integration of MIPs with conductive materials, like graphene oxide. The advantage of the FAPA-MS method lies in the fact that once the adsorption equilibrium is established, the solid polymeric material with the adsorbed analyte is ready for measurement, obviating the need for additional time to wash out the adsorbed analyte.

The time required for measurements assumes a pivotal role, considering the applicability of the presented methodologies across various fields. For instance, the utility of MIP-based detection systems extends to pharmaceutics, where these platforms facilitate the discernment and quantification of biological samples. Therefore, in scenarios demanding swift biological sample analysis, meticulous preparation of the MIP sensor is imperative to enable the required rapid response time. Thus, the ultimate application of the proposed MIPs platform must be taken into account during method preparation.

Conversely, the roster of applicable techniques is steadily expanding. This review concentrates on both conventional and pioneering analytical methodologies, effectively showcasing the broadening landscape of analytical possibilities. This expansion is marked by heightened precision and a consistently growing scope of applications across a wide spectrum of analytes.

## Figures and Tables

**Table 1:** Molecularly imprinted polymer systems used for chromatographic quantification of organic analytes.

Polymer	Analysis Method	LOD	Template	Ref.
Poly(2-vinylpyridine) cross-linked with ethylene glycol dimethacrylate (EGDMA)	Offline MISPE-HPLC-PDA ^1^	2.64 nM	Fenoprofen	[38]
Poly(3-aminopropyltriethoxysilane) cross-linked with tetraethyl orthosilicate and Fe_3_O_4_/SiO_2_ as a support	HPLC-FLD ^2^	1.26 nmol kg^−1^	Zearalenone	[39]
Poly(itaconic acid) cross-linked with EGDMA	HPLC-UV	nd	Sulpiride	[40]
Poly(4-vinylbenzoic acid)	HPLC-UV	nd	Carbamazepine	[41]
Poly(methacrylic acid) cross-linked with EGDMA	Online MISPE-HPLC-UV and HPLC-DAD ^3^	0.04 nM	Climbazole (analyte) Miconazole (dummy template)	[42]
Poly(methacrylic acid) cross-linked with EGDMA and Fe_3_O_4_/SiO_2_ as a support	HPLC-DAD	0.60 nM	Dienestrol	[43]
Composite of mesoporous silica-coated on magnetic graphene oxide and poly(4-vinylbenzoic acid)	Offline MISPE-HPLC-PDA	(No prepolymerization) Sulfadiazine, 0.060 nM; Sulfathiazole, 0.051 nM; Sulfamerazine, 0.045 nM; Sulfamethazine, 0.050 nM; Sulfamethoxazole, 0.051 nM; Sulfadoxine, 0.032 nM (With prepolimeryzation) Sulfadiazine, 0.056 nM; Sulfathiazole, 0.051 nM; Sulfamerazine, 0.045 nM; Sulfamethazine, 0.047 nM; Sulfamethoxazole, 0.047 nM; Sulfadoxine, 0.032 nM	Sulfadiazine, Sulfathiazole, Sulfamerazine, Sulfamethazine, Sulfamethoxazole, Sulfadoxine	[44]
Composite of Poly(dopamine) and Fe_3_O_4_	Offline MISPE-HPLC-UV	1.03 nM	17β-Estradiol	[45]
Composite of Poly(dopamine) and Fe_3_O_4_	Offline MISPE-HPLC-UV	1.87 nM	Tetracycline	[46]
Poly(methacrylic acid) cross-linked with EGDMA	Offline MISPE-LC-MS/MS	3.01 × 10^−7^ nM	Chloramphenicol	[51]
Poly(*p*-vinylbenzoic acid)	LC-MS	0. 034–0.31 ng mL^−1^	Nevirapine, Venlafaxine, Methocarbamol, Carbamazepine, Etilefrine	[53]
Poly(2-vinylpirydine-*co*-methacrylic acid) cross-linked with EGDMA	Offline MISPE-LC-MS	0.3–1.4 ng g^−1^	Bisphenol A	[54]
Poly(methacrylic acid) cross-linked with EGDMA	LC-MS	4.54 nM	Roxithromycin	[52]
Poly(1-vinylimidazole) cross-linked with EGDMA	Offline MISPE-LC-MS/MS	No data	Acid Green 16	[55]
Poly(methacrylic acid) cross-linked with EGDMA	GC-FPD ^4^	150–890 ng L^−1^	Trichlorfon, Dichlorvos, Dimethoate, Imidacloprid, Methamidophos	[58]
Poly(methacrylic acid) cross-linked with EGDMA	Offline MISPE-GC-(μ-ECD)	1 ng g^−1^	Pentachloronitrobenzene	[59]
Polypyrrole	GC-FID ^5^	1.99 nM	Progesterone	[60]
Poly(methacrylic acid) cross-linked with EGDMA	Offline MISPE-GC -(μ-ECD) ^6^	10^−3^ to 5 × 10^−2^ ng	Alachlor, Acetochlor, Pretilachlor, Metolachlor (analytes); Butachlor (dummy template)	[61]
Poly(methacrylic acid) cross-linked with EGDMA	Offline MISPE-GC-MS	4.04 × 10^−3^ nM	N-Nitrosodiphenylamine	[64]
Poly(4-vinylpyridine) cross-linked with EGDMA	Offline MISPE-atmospheric pressure-GC-MS/MS	1–100 ng L^−1^	Naphthalene, Acenaphthylene, Acenaphthene, Fluorene, Phenanthrene, Anthracene, Fluoranthene, Benzo(a)anthracene, Benzo(b)fluoranthene, Benzo(k)fluoranthene, Benzo(a)pyrene, Dibenzo(a,h)anthracene, Benzo(ghi) perylene, Indeno(1,2,3-cd)pyrene (analytes) Toluene (pseudo template)	[65]
Poly(methacrylic acid) cross-linked with EGDMA and poly(methacrylic methacrylate) cross-linked EGDMA	Offline MISPE-GC-MS	0.0302 ng (PBDE-47), 0.0315 ng (PBDE-99)	PBDE-47, PBDE-99 (analytes) Dihydroxydiphenyl ether (dummy template)	[66]
Poly(methacrylic acid) cross-linked with EGDMA	CE-PDA	622 nM	Trichlorfon	[72]
Poly(dopamine)	CE-UV	590 nM	Genistein	[74]
Composite of poly(dopamine-*co*-resorcinol) and Ag and N co-doped zinc oxide supported on activated carbon	CE-MS	0.3 ng g^−1^	Patulin	[75]
Poly(terephthalic acid) cross-linked with EGDMA	CE-DAD	18.77 nM	Atenolol	[73]

^1^ Photodiode array detector, ^2^ fluorescence detector, ^3^ diode array detector, ^4^ flame photometric detector, ^5^ flame ionization detector, ^6^ micro-electron capture detector.

**Table 2:** Molecularly imprinted systems used for quantification of organic analytes by MS measurements.

Polymer	Analysis Method	LOD	Template	Ref.
Polyacrylate	Wooden-tip ESI-MS	10 nM (from water), 50 nM (from fish)	Malachite green	[81]
Poly(methacrylic acid) cross-linked with EGDMA	Wooden-tip ESI-MS	0.003 ng g^−1^ (water), 1.1 ng g^−1^ (honey), 1.9 ng g^−1^ (milk)	Macrolide	[97]
Poly(methacrylic acid) cross-linked with EGDMA	PS-MS	1.57 nM 0.79 nM 0.57 × 10^−5^ nM	Dopamine Sacrosine Butyric acid	[89]
Poly(methacrylic acid) cross-linked with EGDMA	PS-MS	0.89 nM	Cocaine	[93]
Poly(methacrylic acid) cross-linked with EGDMA	PS-MS	3.02 nM	Monouron 2,4,5-T	[96]
Dissolution of nylon-6 in formic acid (8.3 mg mL^−1^)	DI-ESI-MS	9.89 nM 20.1 nM	Cocaine Methamphetamine	[98]

**Table 3:** Molecularly imprinted systems used for quantification of organic analytes via FAPA-MS measurements.

Polymer	Analysis Method	LOD	Template	Ref.
Poly(methacrylic acid)-*co*-(acrylic acid) cross-linked with EGDMA	Microwave argon plasma ionization-MS	0.002 μg to 0.005 μg	Atrazine Pendimethalin Quercetin	[101]
Poly(acrylic acid) cross-linked with EGDMA	FAPA-MS	10 nM (positive), 100 nM (negative)	Quercetin	[102]
Poly(acrylamide)-*co*-(4-vinylpiridine) cross-linked with EGDMA	FAPA-MS	No data	*Trans*-chalcone 2′,4′-dihydroxy- 3-methoxychalcone	[105]
Poly(methacrylic acid) cross-linked with EGDMA	FAPA-MS	10 nM 1000 nM 0.5 × 10^−3^ nM	Nicotine Propyphenazone Methylparaben	[103]
Poly(2-methoxycarbonylpropyl- 2-oxazoline) cross-linked with diethylenetriamine (DETA)	FAPA-MS	1–100 nM	2,4,5-Trichloro- phenoxyacetic acid	[8]
Poly(methyl vinyl ether-*alt*-maleic anhydride) cross-linked with DETA	FAPA-MS	30–600 nM	2,4-Dichlorophenol	[104]

**Table 4:** Molecularly imprinted polymer systems used for electrochemical quantification of organic analytes.

Polymer	Analysis Method	Electrode Type	LOD	Template	Ref.
Poly(methacrylic acid-*co*-4-aminotiophenol	DPV	CPE with Au nanoparticles	0.029 nM	Tetrabromobisphenol-S	[112]
Poly(*m*-phenylenediamine)	DPV	Au SPEL	0.1 nM	Erythromycin	[128]
Polypyrrole/graphene quantum dots	DPV	GCE covered by graphene quantum dots	40 nM	Bisphenol A	[113]
Poly(methacrylic acid) cross-linked with EGDMA	DPV	GCE coated by graphene oxide	0.5 nM	2,4-Dichlorophenol	[114]
Poly(ethylene glycol methacrylate-*co* aniline)	SWV	Carbon SPEL	20 nM	17β-Estradiol	[124]
Poly(Bismarck Brown Y)	EIS	Fluorine-doped tin oxide	160 nM	Uric acid	[132]
Poly(*o*-phenylenediamine)	EIS	GCE coated by graphene oxide	4 × 10^−7^ nM	Testosterone	[133]
Polyimidazole	SWV	GCE coated by Ag nanoparticles and graphene oxide	3.01 × 10^−7^ nM	17β-Estradiol	[121]
Polyacrylamide	DPV; EIS	Au SPEL	7.94 × 10^−4^ nM (DPV), 1.62 × 10^−2^ nM (EIS)	Triclosan	[115]
Poly(*o*-phenylenediamine)	SWV	GCE coated by a zinc oxide nanoparticle/graphene nanoplatelet	40 nM	4-Chlorophenol	[123]
Poly(indole-3-acetic acid)	DPV	GCE and boron-doped diamond electrode	4.9 and 3.2 nM	Cefalexin	[116]
Poly(methacrylic acid) cross-linked with trimethylolpropane trimethacrylate	DPV	Carbon SPEL modified by Au nanoparticles	500 nM	Cyromazine	[117]
Composite of poly(N, N′-methylene-bisacrylamide-*co*-acrylamide) with β-cyclodextrin and reduced graphene oxide	CV, DPV	GCE	8 nM (DPV)	Bisphenol A	[118]
Poly(*o*-phenylenediamine)	EIS	Au SPEL	0.62 nM	Zearalenone	[134]
Poly(methacrylic acid) cross-linked with EGDMA	DPV	GCE	4.08 nM	Chlorpyrifos	[119]
Poly(methacrylic acid) cross-linked with EGDMA	SWV	CPE	0.79 nM	Diazinon	[122]
Poly(*o*-phenylenediamine-*co*-β-cyclodextrin)	DPV	Au electrode modified by Fe-doped porous carbon	0.2 nM	Lomefloxacin	[120]
Polypyrrole	EIS	GCE	850 nM	*N*-nitrosodimethylamine	[129]
Polypyrrole	EIS	Pencil graphite electrode	4.5 nM	Dibutyl phthalate	[130]
Composite of poly(methacrylic acid-*co*-pyrrole) and magnetite nanoparticles	CV, DPV	Carbon SPEL	10^−3^ nM	Tributylin	[131]
Poly(*o*-aminophenol)	EIS	Au-Ag alloy microrod	7.63 × 10^−5^ nM	Dopamine	[126]
Composite of poly(dopamine-*co*-resorcinol) and Ag and N co-doped zinc oxide supported on activated carbon	DPV	GCE	6.7 × 10^−5^ nM	Cypermethrin	[127]
Poly(methacrylic acid) cross-linked with EGDMA	SWV	CPE	0.13 nM	Diazinon	[125]

**Table 5:** Molecularly imprinted systems used for quantification of organic analytes via colorimetric measurements for food safety.

Polymer	Analysis Method	LOD	Template	Ref.
Poly(methacrylic acid) cross-linked with EGDMA	MICS-surface-enhanced Raman spectroscopy (SERS)/colorimetric dual sensor	285.25 nM – 0.28525 mM	Chlorpyrifos	[146]
Poly(methacrylic acid) cross-linked with EGDMA	MICS-surface-enhanced Raman spectroscopy (SERS)/colorimetric dual sensor	0.56 nM	Atrazine	[149]
Poly(methacrylic acid) cross-linked with EGDMA	MICSs colorimetric sensors	100 nM	Phosphorus pesticides	[151]
Poly(methacrylic acid) cross-linked with EGDMA	Ratiometric fluorescence and colorimetric dual-mode	104 nM	Vanillin	[152]
Poly(methacrylic acid) cross-linked with EGDMA	AgNPs colorimetry	25 nM	Caffeine	[153]
Poly(dopamine)	UV-Vis spectroscopy and visual measurements	0.013 mg mL^−1^	Anti-*S. aureus* IgY antibodies	[154]
Poly(*N*-isopropyl acrylamide)	UV-Vis spectroscopy and color intensity measurements	11.83 nM	Glyphosate	[155]
Poly(methacrylic acid) cross-linked with EGDMA	UV-Vis spectroscopy and color change	0.1 nM	Ethyl-*o*-aminobeznoate	[156]

**Table 6:** Molecularly imprinted systems used for quantification of organic analytes via colorimetric measurements in environmental analyses.

Polymer	Analysis Method	LOD	Template	Ref.
Poly(methacrylic acid)-*co*-(acrylamide)	Color change of “radar” pattern and PCA	3.53 µg, 2.42 µg, 4.85 µg, 2.14 µg	2,4,6-trinitrotoluene, 2,6-dinitrotoluene, 2,4-dinitrotoluene, 4-nitrotoluene	[157]
Poly(dopamine)	Intensity of oxidation of TBM	400 nM	Tetracycline	[158]
Poly(styrene)-*co*-(vinyl pyrrolidone) (1:9)	Naked-eye detection	4380 nM	Bisphenol A	[160]
Poly(3-aminopropyltriethoxysilane)-*co*- (tetraethyl orthosilicate)	Intensity of oxidation of TBM	3 pg g^−1^	Tetrabromobisphenol A	[161]
Polu(3-aminopropyltriethoxysilane)-*co*- (phenyltrimethoxysilane)	Color changes via potassium permanganate reduction	0.26 × 10^9^ nM	3-Phenoxybenzaldehyde	[162]
Poly(3-(trimethoxysilyl)propyl methacrylate) cross-linked with EGDMA	Lateral flow immunochromatographic assay	0.0402 nM	Microcystin-LR	[163]

**Table 7:** Molecularly imprinted systems used for quantification of organic analytes via colorimetric measurements in biological sample detection.

Polymer	Analysis Method	LOD	Template	Ref.
Poly(1-ethyl-3-(3-dimethylaminopropyl) carbodiimide)-*co*- *N*-hydroxysuccinimide	Fluorescence and visualization	8.33 × 10^−6^ nM (fluorescence), 2.08 × 10^−3^ nM (visualization)	Enterovirus 71 (EV71)	[164]
Poly(methacrylic acid) cross-linked with *N,N*′-methylene bisacrylamide (BIS)	Laser pointer and visualization	50 nM	L-Kynurenine	[166]
Poly(3-acrylamidophenylboronic acid)-*co*- (2-hydroxy-2-methylpropiophenone) cross-linked with BIS	Intensity of oxidation of TBM	1.32 ng mL^−1^	Glycoprotein	[167]
Poly(dopamine)	Intensity of oxidation of TBM	0.0278 nM	Thrombin	[168]
Poly(norepinephrine)	Colorimetric indirect competitive bioassay	0.227 nM	Gonadotropin	[169]
Poly(2,4-difluoro-3-formylphenylboronic acid)	Visually structure color changes	0.3 nM	Horseradish peroxidase	[170]
Poly(4-vinylphenylboronate)	A cascaded catalytic system with glucose oxidase	400 nM	β-D-glucose	[171]

**Table 8:** Molecularly imprinted systems used for quantification of organic analytes via colorimetric measurements in drug detection and medical treatment.

Polymer	Analysis Method	LOD	Template	Ref.
Poly(methacrylic acid)-*co*-(acrylamide) cross-linked with EGDMA	Dye displacement and UV-Vis measurements	No data	Amoxicillin and dyes	[173]
Poly(methacrylic acid) cross-linked with EGDMA	Dye displacement and UV-Vis measurements	50,000 nM	2-methoxiphenidine and dyes	[174]
Poly(methacrylic acid)-*co*- (acetazolamide) cross-linked with EGDMA	Smartphone-based detection	30 nM	Acetazolamide	[175]

**Table 9:** Molecularly imprinted systems used for quantification of organic analytes by plasmonic resonance measurements.

Polymer	Analysis Method	LOD	Template	Ref.
Poly(vinylbenzyl)trimethylammonium chloride-co-1H,1H,2H,2H-perfluoroalkyl acrylate cross-linked with EGDMA	SPR sensor based on a D-shaped POF	0.13 ppb	Ammonium perfluorooctanoate	[186]
Poly(vinyl alcohol) cross-linked with EGDMA	SPR	3.3 nM	Atrazine	[187]
Poly(methacrylic acid) cross-linked with divinylbenzene (DVB)	SPR sensor based on a D-shaped POF	41.53 nM	2-Furaldhyde	[188]
Poly(methacrylamide-*co*-vinyl trimethoxysilane-*co*-tetrahydroxilane)	SPR	0.073 nM	Amoxicillin	[189]
Poly(acrylamide-*co*-*N*-t-butylacrylamide-*co*- 2-hydroxyethyl methacrylate	SPR sensor based on a D-shaped POF	51,000 nM	Subunit 1 of the SARS-CoV-2 spike protein	[190]
Poly(2-hydroxyethyl methacrylate-*co*- *N*-(hydroxymethyl)acrylamide solution-*co*-*N*-isopropylacrylamide-*co*-acrylamide)	SPR biosensor	0.23 nM	Secreted bacterial factor	[191]
Poly(vinylbenzyl)trimethylammonium chloride-co-1H,1H,2H,2H-perfluoroalkyl acrylate cross-linked with EGDMA	(1) D-shaped POF with MIP, (2) SPR sensor based on a D-shaped POF	0.81 ppt	Perfluorooctanoic acid	[201]
Poly(N-isopropylacrylamide-*co*-methacrylic acid)	LSPR	No data	Lysozyme and Lactoferrin	[204]
Poly(methacrylic acid-vinylbenzyl trimethylammonium chloride) cross-linked with EGDMA	LSPR	No data	Polyphenol	[206]

**Table 10:** Molecularly imprinted systems used for quantification of organic analytes via fluorescence measurements.

Polymer	Analysis Method	LOD	Template	Ref.
Poly(3-aminopropyl triethoxysilan) cross-linked by tetraethoxysilane	Paper-based fluorescence sensor	0.274 nM	Malachite Green	[220]
Polymerizable anion receptors containing a urea-type binding sites cross-linked with EGDMA	Fluoride-imprinted polymeric optical sensor	11,300 nM	Fluoride ion	[221]
Poly(methacrylic acid) cross-linked with EGDMA	Sensitive fluorometric method	3.64 nM	Quercetin	[224]
Poly(methacrylic acid) cross-linked with EGDMA	Fluorescence test strip	1.46 × 10^6^ nM	Tyramine	[226]
Poly(3-aminopropyl triethoxysilan) cross-linked with tetraethoxysilane	Fluorescence quenching of quantum dots	350 nM	Chloramphenicol	[228]
Poly(3-aminopropyl triethoxysilan) cross-linked with tetraethoxysilane	Fluorescence quenching effect	9.98 × 10^−3^ nM	Microcystin-LR	[229]
Poly(3-aminopropyl triethoxysilan) cross-linked with tetraethoxysilane	Fluorescence probe of polypyrrole and quantum dots	0.143 nM	Ampicillin	[230]

## Data Availability

Not applicable.

## References

[1] Lusina A., Cegłowski M. (2022). Molecularly Imprinted Polymers as State-of-the-Art Drug Carriers in Hydrogel Transdermal Drug Delivery Applications. Polymers.

[2] Zhang Z., Li H., Liao H., Nie L., Yao S. (2005). Influence of cross-linkers’ amount on the performance of the piezoelectric sensor modified with molecularly imprinted polymers. Sensor. Actuat. B-Chem..

[3] Basak S., Venkatram R., Singhal R.S. (2022). Recent advances in the application of molecularly imprinted polymers (MIPs) in food analysis. Food Control.

[4] Selvolini G., Marrazza G. (2017). MIP-based sensors: Promising new tools for cancer biomarker determination. Sensors.

[5] Mostafa A.M., Barton S.J., Wren S.P., Barker J. (2021). Review on molecularly imprinted polymers with a focus on their application to the analysis of protein biomarkers. TrAC Trends Anal. Chem..

[6] Lok C., Son R. (2009). Application of molecularly imprinted polymers in food sample analysis—A perspective. Int. Food Res. J..

[7] Chen L., Wang X., Lu W., Wu X., Li J. (2016). Molecular imprinting: Perspectives and applications. Chem. Soc. Rev..

[8] Cegłowski M., Marien Y.W., Smeets S., De Smet L., D’hooge D.R., Schroeder G., Hoogenboom R. (2021). Molecularly Imprinted Polymers with Enhanced Selectivity Based on 4-(Aminomethyl) pyridine-Functionalized Poly (2-oxazoline) s for Detecting Hazardous Herbicide Contaminants. Chem. Mater..

[9] Unger C., Lieberzeit P.A. (2021). Molecularly imprinted thin film surfaces in sensing: Chances and challenges. React. Funct. Polym..

[10] Lowdon J.W., Diliën H., Singla P., Peeters M., Cleij T.J., van Grinsven B., Eersels K. (2020). MIPs for commercial application in low-cost sensors and assays–An overview of the current status quo. Sens. Actuators B Chem..

[11] Battal D., Akgönüllü S., Yalcin M.S., Yavuz H., Denizli A. (2018). Molecularly imprinted polymer based quartz crystal microbalance sensor system for sensitive and label-free detection of synthetic cannabinoids in urine. Biosens. Bioelectron..

[12] Chunta S., Suedee R., Lieberzeit P.A. (2018). High-density lipoprotein sensor based on molecularly imprinted polymer. Anal. Bioanal. Chem..

[13] Altintas Z. (2018). Surface plasmon resonance based sensor for the detection of glycopeptide antibiotics in milk using rationally designed nanoMIPs. Sci. Rep..

[14] Canfarotta F., Smolinska-Kempisty K., Piletsky S. (2017). Replacement of antibodies in pseudo-ELISAs: Molecularly imprinted nanoparticles for vancomycin detection. Synth. Antibodies: Methods Protoc..

[15] D’Aurelio R., Chianella I., Goode J.A., Tothill I.E. (2020). Molecularly imprinted nanoparticles based sensor for cocaine detection. Biosensors.

[16] Huang X., Xia L., Li G. (2023). Recent Progress of Molecularly Imprinted Optical Sensors. Chemosensors.

[17] Ayivi R.D., Adesanmi B.O., McLamore E.S., Wei J., Obare S.O. (2023). Molecularly Imprinted Plasmonic Sensors as Nano-Transducers: An Effective Approach for Environmental Monitoring Applications. Chemosensors.

[18] Pilvenyte G., Ratautaite V., Boguzaite R., Ramanavicius A., Viter R., Ramanavicius S. (2023). Molecularly Imprinted Polymers for the Determination of Cancer Biomarkers. Int. J. Mol. Sci..

[19] Nageib A.M., Halim A.A., Nordin A.N., Ali F. (2023). Recent Applications of Molecularly Imprinted Polymers (MIPs) on Screen-Printed Electrodes for Pesticide Detection. J. Electrochem. Sci. Technol.

[20] Bélanger J.M.R., Jocelyn Paré J.R., Sigouin M., Paré J.R.J., Bélanger J.M.R. (1997). Chapter 2 High performance liquid chromatography (HPLC): Principles and applications. Techniques and Instrumentation in Analytical Chemistry.

[21] Bird I.M. (1989). High Performance Liquid Chromatography: Principles And Clinical Applications. BMJ Br. Med. J..

[22] Dorsey J.G., Cooper W.T., Siles B.A., Foley J.P., Barth H.G. (1996). Liquid Chromatography:  Theory and Methodology. Anal. Chem..

[23] Kalász H., Báthori M., Valkó K.L., Valkó K.L. (2020). Chapter 10—Basis and pharmaceutical applications of thin-layer chromatography. Handbook of Analytical Separations.

[24] Nováková L., Svoboda P., Pavlík J., Fanali S., Haddad P.R., Poole C.F., Riekkola M.-L. (2017). Chapter 29—Ultra-high performance liquid chromatography. Liquid Chromatography.

[25] Stoll D.R., Carr P.W. (2017). Two-Dimensional Liquid Chromatography: A State of the Art Tutorial. Anal. Chem..

[26] Lhotská I., Holznerová A., Solich P., Šatínský D. (2017). Critical comparison of the on-line and off-line molecularly imprinted solid-phase extraction of patulin coupled with liquid chromatography. J. Sep. Sci..

[27] Wan Q., Liu H., Deng Z., Bu J., Li T., Yang Y., Zhong S. (2021). A critical review of molecularly imprinted solid phase extraction technology. J. Polym. Res..

[28] Fresco-Cala B., Batista A.D., Cárdenas S. (2020). Molecularly Imprinted Polymer Micro- and Nano-Particles: A review. Molecules.

[29] Moein M.M., Javanbakht M., Akbari-adergani B. (2014). Molecularly imprinted polymer cartridges coupled on-line with high performance liquid chromatography for simple and rapid analysis of human insulin in plasma and pharmaceutical formulations. Talanta.

[30] Liu Y., Dang X., Chen H. (2023). A molecularly imprinted polymer monolithic column with dual template and bifunctional monomers for selective extraction and simultaneous determination of eight phenolics from polycarbonate cups. Anal. Chim. Acta.

[31] Orowitz T.E., Ana Sombo P., Rahayu D., Hasanah A.N. (2020). Microsphere Polymers in Molecular Imprinting: Current and Future Perspectives. Molecules.

[32] Boysen R.I. (2019). Advances in the development of molecularly imprinted polymers for the separation and analysis of proteins with liquid chromatography. J. Sep. Sci..

[33] Zhang B., Zhao J., Sha B., Xian M. (2012). Selective solid-phase extraction using molecularly imprinted polymers for the analysis of norfloxacin in fish. Anal. Methods.

[34] Metwally M.G., Benhawy A.H., Khalifa R.M., El Nashar R.M., Trojanowicz M. (2021). Application of Molecularly Imprinted Polymers in the Analysis of Waters and Wastewaters. Molecules.

[35] Azizi A., Bottaro C.S. (2020). A critical review of molecularly imprinted polymers for the analysis of organic pollutants in environmental water samples. J. Chromatogr. A.

[36] Hu T., Chen R., Wang Q., He C., Liu S. (2021). Recent advances and applications of molecularly imprinted polymers in solid-phase extraction for real sample analysis. J. Sep. Sci..

[37] Gama M.R., Bottoli C.B.G. (2017). Molecularly imprinted polymers for bioanalytical sample preparation. J. Chromatogr. B.

[38] Mbhele Z.E., Ncube S., Madikizela L.M. (2018). Synthesis of a molecularly imprinted polymer and its application in selective extraction of fenoprofen from wastewater. Environ. Sci. Pollut. Res..

[39] Fu H., Xu W., Wang H., Liao S., Chen G. (2020). Preparation of magnetic molecularly imprinted polymers for the identification of zearalenone in grains. Anal. Bioanal. Chem..

[40] Zhang W., She X., Wang L., Fan H., Zhou Q., Huang X., Tang J.Z. (2017). Preparation, Characterization and Application of a Molecularly Imprinted Polymer for Selective Recognition of Sulpiride. Materials.

[41] He Q., Liang J.-J., Chen L.-X., Chen S.-L., Zheng H.-L., Liu H.-X., Zhang H.-J. (2020). Removal of the environmental pollutant carbamazepine using molecular imprinted adsorbents: Molecular simulation, adsorption properties, and mechanisms. Water Res..

[42] Sun X., Wang M., Peng J., Yang L., Wang X., Wang F., Zhang X., Wu Q., Chen R., Chen J. (2019). Dummy molecularly imprinted solid phase extraction of climbazole from environmental water samples. Talanta.

[43] He X.-P., Lian Z.-R., Tan L.-J., Wang J.-T. (2016). Preparation and characterization of magnetic molecularly imprinted polymers for selective trace extraction of dienestrol in seawater. J. Chromatogr. A.

[44] Fan Y., Zeng G., Ma X. (2020). Effects of prepolymerization on surface molecularly imprinted polymer for rapid separation and analysis of sulfonamides in water. J. Colloid Interface Sci..

[45] Wang Y., Zhao W., Gao R., Hussain S., Hao Y., Tian J., Chen S., Feng Y., Zhao Y., Qu Y. (2022). Preparation of lightweight daisy-like magnetic molecularly imprinted polymers via etching synergized template immobilization for enhanced rapid detection of trace 17β-estradiol. J. Hazard. Mater..

[46] Wang Y., Xu Y., Gao R., Tian X., Heinlein J., Hussain S., Pfefferle L.D., Chen X., Zhang X., Hao Y. (2022). Strategic design and fabrication of lightweight sesame ball-like hollow double-layer hybrid magnetic molecularly imprinted nanomaterials for the highly specific separation and recovery of tetracycline from milk. Green Chem..

[47] Stokvis E., Rosing H., Beijnen J.H. (2005). Liquid chromatography-mass spectrometry for the quantitative bioanalysis of anticancer drugs. Mass Spectrom. Rev..

[48] McMaster M.C. (2005). LC/MS: A Practical User’s Guide.

[49] Jemal M., Xia Y.-Q. (2006). LC-MS Development Strategies for Quantitative Bioanalysis. Curr. Drug Metab..

[50] Xu R.N., Fan L., Rieser M.J., El-Shourbagy T.A. (2007). Recent advances in high-throughput quantitative bioanalysis by LC–MS/MS. J. Pharm. Biomed. Anal..

[51] Zhao F., She Y., Zhang C., Wang S., Du X., Jin F., Jin M., Shao H., Zheng L., Wang J. (2017). Selective Determination of Chloramphenicol in Milk Samples by the Solid-Phase Extraction Based on Dummy Molecularly Imprinted Polymer. Food Anal. Methods.

[52] Ding J., Zhang F., Zhang X., Wang L., Wang C., Zhao Q., Xu Y., Ding L., Ren N. (2016). Determination of roxithromycin from human plasma samples based on magnetic surface molecularly imprinted polymers followed by liquid chromatography-tandem mass spectromer. J. Chromatogr. B.

[53] Khulu S., Ncube S., Kgame T., Mavhunga E., Chimuka L. (2022). Synthesis, characterization and application of a molecularly imprinted polymer as an adsorbent for solid-phase extraction of selected pharmaceuticals from water samples. Polym. Bull..

[54] Maragou N.C., Thomaidis N.S., Theodoridis G.A., Lampi E.N., Koupparis M.A. (2020). Determination of bisphenol A in canned food by microwave assisted extraction, molecularly imprinted polymer-solid phase extraction and liquid chromatography-mass spectrometry. J. Chromatogr. B.

[55] Foguel M.V., Pedro N.T.B., Wong A., Khan S., Zanoni M.V.B., Sotomayor M. (2017). Synthesis and evaluation of a molecularly imprinted polymer for selective adsorption and quantification of Acid Green 16 textile dye in water samples. Talanta.

[56] Wang Z., Jocelyn Paré J.R., Paré J.R.J., Bélanger J.M.R. (1997). Chapter 3 Gas chromatography (GC): Principles and applications. Techniques and Instrumentation in Analytical Chemistry.

[57] McNair H.M., Miller J.M., Snow N.H. (2019). Basic Gas Chromatography.

[58] Chen J., Zhang W.-t., Shu Y., Ma X.-h., Cao X.-y. (2017). Detection of Organophosphorus Pesticide Residues in Leaf Lettuce and Cucumber Through Molecularly Imprinted Solid-Phase Extraction Coupled to Gas Chromatography. Food Anal. Methods.

[59] Xu X., Liang S. (2019). Molecularly imprinted solid-phase extraction method for the gas chromatographic analysis of organochlorine fungicides in ginseng. J. Sep. Sci..

[60] Nezhadali A., Es’haghi Z., Khatibi A. (2016). Selective extraction of progesterone hormones from environmental and biological samples using a polypyrrole molecularly imprinted polymer and determination by gas chromatography. Anal. Methods.

[61] Wang Y., Jin X., Zhao D., Guo X., Li R. (2015). Molecularly imprinted solid-phase extraction coupled with gas chromatography for the determination of four chloroacetamide herbicides in soil. Anal. Methods.

[62] Sneddon J., Masuram S., Richert J.C. (2007). Gas Chromatography-Mass Spectrometry-Basic Principles, Instrumentation and Selected Applications for Detection of Organic Compounds. Anal. Lett..

[63] Lasáková M., Jandera P. (2009). Molecularly imprinted polymers and their application in solid phase extraction. J. Sep. Sci..

[64] Li Z., Qian Z., Hu S., Gong T., Xian Q. (2018). Molecularly imprinted solid phase extraction coupled with gas chromatography-mass spectrometry for determination of N-Nitrosodiphenylamine in water samples. Chemosphere.

[65] Shahhoseini F., Azizi A., Egli S.N., Bottaro C.S. (2020). Single-use porous thin film extraction with gas chromatography atmospheric pressure chemical ionization tandem mass spectrometry for high-throughput analysis of 16 PAHs. Talanta.

[66] Marć M., Panuszko A., Namieśnik J., Wieczorek P.P. (2018). Preparation and characterization of dummy-template molecularly imprinted polymers as potential sorbents for the recognition of selected polybrominated diphenyl ethers. Anal. Chim. Acta.

[67] Xu X., Duhoranimana E., Zhang X. (2017). Preparation and characterization of magnetic molecularly imprinted polymers for the extraction of hexamethylenetetramine in milk samples. Talanta.

[68] Kemp G. (1998). Capillary electrophoresis: A versatile family of analytical techniques. Biotechnol. Appl. Biochem..

[69] Gackowski M., Przybylska A., Kruszewski S., Koba M., Mądra-Gackowska K., Bogacz A. (2021). Recent Applications of Capillary Electrophoresis in the Determination of Active Compounds in Medicinal Plants and Pharmaceutical Formulations. Molecules.

[70] Rutkowska M., Namieśnik J., Marć M. (2019). Chapter Nine—Molecularly imprinted polymers applied in capillary electrochromatography and electrophoresis techniques. Comprehensive Analytical Chemistry.

[71] Lobato A., Pereira E.A., Gonçalves L.M. (2021). Combining capillary electromigration with molecular imprinting techniques towards an optimal separation and determination. Talanta.

[72] Li J., Lu J., Qiao X., Xu Z. (2017). A study on biomimetic immunoassay-capillary electrophoresis method based on molecularly imprinted polymer for determination of trace trichlorfon residue in vegetables. Food Chem..

[73] da Silva A.T.M., Pires B.C., Dinali L.A.F., Maia A.C.F.C., dos Santos C.J., Sanches C., Borges W.d.S., Borges K.B. (2021). Terephthalic acid-based magnetic molecularly imprinted polymer for enantioselective capillary electrophoresis determination of atenolol in human plasma. Sep. Purif. Technol..

[74] Bezdekova J., Vlcnovska M., Zemankova K., Bacova R., Kolackova M., Lednicky T., Pribyl J., Richtera L., Vanickova L., Adam V. (2020). Molecularly imprinted polymers and capillary electrophoresis for sensing phytoestrogens in milk. J. Dairy Sci..

[75] Moreno-González D., Jáč P., Riasová P., Nováková L. (2021). In-line molecularly imprinted polymer solid phase extraction-capillary electrophoresis coupled with tandem mass spectrometry for the determination of patulin in apple-based food. Food Chem..

[76] Fenn J.B., Mann M., Meng C.K., Wong S.F., Whitehouse C.M. (1989). Electrospray ionization for mass spectrometry of large biomolecules. Science.

[77] Aksenov A.A., da Silva R., Knight R., Lopes N.P., Dorrestein P.C. (2017). Global chemical analysis of biology by mass spectrometry. Nat. Rev. Chem..

[78] Hu B., So P.-K., Chen H., Yao Z.-P. (2011). Electrospray ionization using wooden tips. Anal. Chem..

[79] Hu B., Yao Z.-P. (2022). Electrospray ionization mass spectrometry with wooden tips: A review. Anal. Chim. Acta.

[80] Peacock P.M., Zhang W.-J., Trimpin S. (2017). Advances in ionization for mass spectrometry. Anal. Chem..

[81] Huang Y., Ma Y., Hu H., Guo P., Miao L., Yang Y., Zhang M. (2017). Rapid and sensitive detection of trace malachite green and its metabolite in aquatic products using molecularly imprinted polymer-coated wooden-tip electrospray ionization mass spectrometry. RSC Adv..

[82] Srivastava S., Sinha R., Roy D. (2004). Toxicological effects of malachite green. Aquat. Toxicol..

[83] Stolker A., Zuidema T., Nielen M. (2007). Residue analysis of veterinary drugs and growth-promoting agents. TrAC Trends Anal. Chem..

[84] Damon D.E., Davis K.M., Moreira C.R., Capone P., Cruttenden R., Badu-Tawiah A.K. (2016). Direct biofluid analysis using hydrophobic paper spray mass spectrometry. Anal. Chem..

[85] Santos H., Martins R., Soares D., Chaves A. (2020). Molecularly imprinted polymers for miniaturized sample preparation techniques: Strategies for chromatographic and mass spectrometry methods. Anal. Methods.

[86] Ferreira C.R., Yannell K.E., Jarmusch A.K., Pirro V., Ouyang Z., Cooks R.G. (2016). Ambient ionization mass spectrometry for point-of-care diagnostics and other clinical measurements. Clin. Chem..

[87] Gómez-Ríos G.A., Reyes-Garcés N., Bojko B., Pawliszyn J. (2016). Biocompatible solid-phase microextraction nanoelectrospray ionization: An unexploited tool in bioanalysis. Anal. Chem..

[88] Espy R.D., Muliadi A.R., Ouyang Z., Cooks R.G. (2012). Spray mechanism in paper spray ionization. Int. J. Mass Spectrom..

[89] Mendes T.P., Pereira I., Ferreira M.R., Chaves A.R., Vaz B.G. (2017). Molecularly imprinted polymer-coated paper as a substrate for highly sensitive analysis using paper spray mass spectrometry: Quantification of metabolites in urine. Anal. Methods.

[90] Lu H., Yu J., Wang J., Wu L., Xiao H., Gao R. (2016). Simultaneous quantification of neuroactive dopamine serotonin and kynurenine pathway metabolites in gender-specific youth urine by ultra performance liquid chromatography tandem high resolution mass spectrometry. J. Pharm. Biomed. Anal..

[91] Mazzu-Nascimento T., Leão P.A.G.C., Catai J.R., Morbioli G.G., Carrilho E. (2016). Towards low-cost bioanalytical tools for sarcosine assays for cancer diagnostics. Anal. Methods.

[92] Imai K., Ochiai K. (2013). Effect of microbial coinfection with HIV-1 and butyric acid-producing anaerobic bacteria on AIDS progression. J. Oral Biosci..

[93] Tavares L.S., Carvalho T.C., Romão W., Vaz B.G., Chaves A.R. (2017). Paper spray tandem mass spectrometry based on molecularly imprinted polymer substrate for cocaine analysis in oral fluid. J. Am. Soc. Mass Spectrom..

[94] Frederick D.L. (2012). Toxicology testing in alternative specimen matrices. Clin. Lab. Med..

[95] Montesano C., Simeoni M.C., Curini R., Sergi M., Lo Sterzo C., Compagnone D. (2015). Determination of illicit drugs and metabolites in oral fluid by microextraction on packed sorbent coupled with LC-MS/MS. Anal. Bioanal. Chem..

[96] Pereira I., Rodrigues M.F., Chaves A.R., Vaz B.G. (2018). Molecularly imprinted polymer (MIP) membrane assisted direct spray ionization mass spectrometry for agrochemicals screening in foodstuffs. Talanta.

[97] Liu Y., Yang Q., Chen X., Song Y., Wu Q., Yang Y., He L. (2019). Sensitive analysis of trace macrolide antibiotics in complex food samples by ambient mass spectrometry with molecularly imprinted polymer-coated wooden tips. Talanta.

[98] Díaz-Liñán M., García-Valverde M., Lucena R., Cárdenas S., López-Lorente A. (2021). Dual-template molecularly imprinted paper for the determination of drugs of abuse in saliva samples by direct infusion mass spectrometry. Microchem. J..

[99] Huang M.-Z., Cheng S.-C., Cho Y.-T., Shiea J. (2011). Ambient ionization mass spectrometry: A tutorial. Anal. Chim. Acta.

[100] Harris G.A., Galhena A.S., Fernandez F.M. (2011). Ambient sampling/ionization mass spectrometry: Applications and current trends. Anal. Chem..

[101] Guć M., Reszke E., Cegłowski M., Schroeder G. (2019). The application of the microwave plasma ionization source in ambient mass spectrometry. Plasma Chem. Plasma Process..

[102] Guć M., Schroeder G. (2019). Application of molecularly imprinted polymers (MIP) and magnetic molecularly imprinted polymers (mag-MIP) to selective analysis of quercetin in flowing atmospheric-pressure afterglow mass spectrometry (FAPA-MS) and in electrospray ionization mass spectrometry (ESI-MS). Molecules.

[103] Cegłowski M., Smoluch M., Reszke E., Silberring J., Schroeder G. (2017). Molecularly imprinted polymers as selective adsorbents for ambient plasma mass spectrometry. Anal. Bioanal. Chem..

[104] Bogdanowicz N., Lusina A., Nazim T., Cegłowski M. (2023). Rapid quantification of 2, 4-dichlorophenol in river water samples using molecularly imprinted polymers coupled to ambient plasma mass spectrometry. J. Hazard. Mater..

[105] Pawlaczyk M., Guc M., Schroeder G. (2021). Adsorption and selectivity studies of direct and magnetite-cored molecularly imprinted polymers (MIPs and magMIPs) towards chosen chalcones investigated with various analytical methods. RSC Adv..

[106] Elugoke S.E., Adekunle A.S., Fayemi O.E., Akpan E.D., Mamba B.B., Sherif E.-S.M., Ebenso E.E. (2021). Molecularly imprinted polymers (MIPs) based electrochemical sensors for the determination of catecholamine neurotransmitters—Review. Electrochem. Sci. Adv..

[107] Manikandan R., Deepa P.N., Narayanan S.S. (2020). Simultaneous electrochemical determination of adenine and guanine using poly 2-naphthol orange film–modified electrode. Ionics.

[108] Ribeiro J.A., Fernandes P.M.V., Pereira C.M., Silva F. (2016). Electrochemical sensors and biosensors for determination of catecholamine neurotransmitters: A review. Talanta.

[109] Chen A., Shah B. (2013). Electrochemical sensing and biosensing based on square wave voltammetry. Anal. Methods.

[110] Elfadil D., Lamaoui A., Della Pelle F., Amine A., Compagnone D. (2021). Molecularly Imprinted Polymers Combined with Electrochemical Sensors for Food Contaminants Analysis. Molecules.

[111] Shao Y., Duan J., Wang M., Cao J., She Y., Cao Z., Li G., Jin F., Wang J., Abd El-Aty A.M. (2023). Application of Molecularly Imprinted Electrochemical Biomimetic Sensors for Detecting Small Molecule Food Contaminants. Polymers.

[112] Sarpong K.A., Zhang K., Luan Y., Cao Y., Xu W. (2020). Development and application of a novel electrochemical sensor based on AuNPS and difunctional monomer-MIPs for the selective determination of Tetrabromobisphenol-S in water samples. Microchem. J..

[113] Tan F., Cong L., Li X., Zhao Q., Zhao H., Quan X., Chen J. (2016). An electrochemical sensor based on molecularly imprinted polypyrrole/graphene quantum dots composite for detection of bisphenol A in water samples. Sens. Actuators B Chem..

[114] Liang Y., Yu L., Yang R., Li X., Qu L., Li J. (2017). High sensitive and selective graphene oxide/molecularly imprinted polymer electrochemical sensor for 2,4-dichlorophenol in water. Sens. Actuators B Chem..

[115] Motia S., Tudor I.A., Ribeiro P.A., Raposo M., Bouchikhi B., El Bari N. (2019). Electrochemical sensor based on molecularly imprinted polymer for sensitive triclosan detection in wastewater and mineral water. Sci. Total Environ..

[116] Feier B., Blidar A., Pusta A., Carciuc P., Cristea C. (2019). Electrochemical Sensor Based on Molecularly Imprinted Polymer for the Detection of Cefalexin. Biosensors.

[117] Peng S., Wang A., Lian Y., Jia J., Ji X., Yang H., Li J., Yang S., Liao J., Zhou S. (2022). Technology for Rapid Detection of Cyromazine Residues in Fruits and Vegetables: Molecularly Imprinted Electrochemical Sensors. Biosensors.

[118] Ali H., Mukhopadhyay S., Jana N.R. (2019). Selective electrochemical detection of bisphenol A using a molecularly imprinted polymer nanocomposite. New J. Chem..

[119] Xu W., Wang Q., Huang W., Yang W. (2017). Construction of a novel electrochemical sensor based on molecularly imprinted polymers for the selective determination of chlorpyrifos in real samples. J. Sep. Sci..

[120] Li J., Huang X., Ma J., Wei S., Zhang H. (2020). A novel electrochemical sensor based on molecularly imprinted polymer with binary functional monomers at Fe-doped porous carbon decorated Au electrode for the sensitive detection of lomefloxacin. Ionics.

[121] Biyana Regasa M., Nyokong T. (2022). Synergistic recognition and electrochemical sensing of 17β-Estradiol using ordered molecularly imprinted polymer-graphene oxide-silver nanoparticles composite films. J. Electroanal. Chem..

[122] Motaharian A., Motaharian F., Abnous K., Hosseini M.R.M., Hassanzadeh-Khayyat M. (2016). Molecularly imprinted polymer nanoparticles-based electrochemical sensor for determination of diazinon pesticide in well water and apple fruit samples. Anal. Bioanal. Chem..

[123] Al-Ammari R.H., Ganash A.A., Salam M.A. (2019). Electrochemical molecularly imprinted polymer based on zinc oxide/graphene/poly(o-phenylenediamine) for 4-chlorophenol detection. Synth. Met..

[124] Lahcen A.A., Baleg A.A., Baker P., Iwuoha E., Amine A. (2017). Synthesis and electrochemical characterization of nanostructured magnetic molecularly imprinted polymers for 17-β-Estradiol determination. Sens. Actuators B Chem..

[125] Khadem M., Faridbod F., Norouzi P., Rahimi Foroushani A., Ganjali M.R., Shahtaheri S.J., Yarahmadi R. (2017). Modification of Carbon Paste Electrode Based on Molecularly Imprinted Polymer for Electrochemical Determination of Diazinon in Biological and Environmental Samples. Electroanalysis.

[126] Li Y., Song H., Zhang L., Zuo P., Ye B.-c., Yao J., Chen W. (2016). Supportless electrochemical sensor based on molecularly imprinted polymer modified nanoporous microrod for determination of dopamine at trace level. Biosens. Bioelectron..

[127] Li Y., Zhang L., Dang Y., Chen Z., Zhang R., Li Y., Ye B.-C. (2019). A robust electrochemical sensing of molecularly imprinted polymer prepared by using bifunctional monomer and its application in detection of cypermethrin. Biosens. Bioelectron..

[128] Ayankojo A.G., Reut J., Ciocan V., Öpik A., Syritski V. (2020). Molecularly imprinted polymer-based sensor for electrochemical detection of erythromycin. Talanta.

[129] Cetó X., Saint C.P., Chow C.W.K., Voelcker N.H., Prieto-Simón B. (2016). Electrochemical detection of N-nitrosodimethylamine using a molecular imprinted polymer. Sens. Actuators B Chem..

[130] Bolat G., Yaman Y.T., Abaci S. (2019). Molecularly imprinted electrochemical impedance sensor for sensitive dibutyl phthalate (DBP) determination. Sens. Actuators B Chem..

[131] Zamora-Gálvez A., Mayorga-Matinez C.C., Parolo C., Pons J., Merkoçi A. (2017). Magnetic nanoparticle-molecular imprinted polymer: A new impedimetric sensor for tributyltin detection. Electrochem. Commun..

[132] Trevizan H.F., Olean-Oliveira A., Cardoso C.X., Teixeira M.F.S. (2021). Development of a molecularly imprinted polymer for uric acid sensing based on a conductive azopolymer: Unusual approaches using electrochemical impedance/capacitance spectroscopy without a soluble redox probe. Sens. Actuators B Chem..

[133] Liu W., Ma Y., Sun G., Wang S., Deng J., Wei H. (2017). Molecularly imprinted polymers on graphene oxide surface for EIS sensing of testosterone. Biosens. Bioelectron..

[134] Radi A.-E., Eissa A., Wahdan T. (2020). Molecularly Imprinted Impedimetric Sensor for Determination of Mycotoxin Zearalenone. Electroanalysis.

[135] Nishitani S., Sakata T. (2018). Potentiometric Adsorption Isotherm Analysis of a Molecularly Imprinted Polymer Interface for Small-Biomolecule Recognition. ACS Omega.

[136] Sakata T. (2019). Biologically Coupled Gate Field-Effect Transistors Meet in Vitro Diagnostics. ACS Omega.

[137] Kaisti M. (2017). Detection principles of biological and chemical FET sensors. Biosens. Bioelectron..

[138] Yang H., Nishitani S., Sakata T. (2018). Potentiometric Langmuir Isotherm Analysis of Histamine-Selective Molecularly Imprinted Polymer-Based Field-Effect Transistor. ECS J. Solid State Sci. Technol..

[139] Kajisa T., Li W., Michinobu T., Sakata T. (2018). Well-designed dopamine-imprinted polymer interface for selective and quantitative dopamine detection among catecholamines using a potentiometric biosensor. Biosens. Bioelectron..

[140] Nishitani S., Kajisa T., Sakata T. (2017). Development of molecularly imprinted polymer-based field effect transistor for sugar chain sensing. Jpn. J. Appl. Phys..

[141] Kajisa T., Sakata T. (2018). Molecularly Imprinted Artificial Biointerface for an Enzyme-Free Glucose Transistor. ACS Appl. Mater. Interfaces.

[142] Bartold K., Iskierko Z., Borowicz P., Noworyta K., Lin C.-Y., Kalecki J., Sharma P.S., Lin H.-Y., Kutner W. (2022). Molecularly imprinted polymer-based extended-gate field-effect transistor (EG-FET) chemosensor for selective determination of matrix metalloproteinase-1 (MMP-1) protein. Biosens. Bioelectron..

[143] Iskierko Z., Checinska A., Sharma P.S., Golebiewska K., Noworyta K., Borowicz P., Fronc K., Bandi V., D’Souza F., Kutner W. (2017). Molecularly imprinted polymer based extended-gate field-effect transistor chemosensors for phenylalanine enantioselective sensing. J. Mater. Chem. C.

[144] Monogarova O., Oskolok K., Apyari V. (2018). Colorimetry in chemical analysis. J. Anal. Chem..

[145] Wang S., Xu S., Zhou Q., Liu Z., Xu Z. (2023). State-of-the-art molecular imprinted colorimetric sensors and their on-site inspecting applications. J. Sep. Sci..

[146] Feng S., Hu Y., Ma L., Lu X. (2017). Development of molecularly imprinted polymers-surface-enhanced Raman spectroscopy/colorimetric dual sensor for determination of chlorpyrifos in apple juice. Sens. Actuators B Chem..

[147] Anirudhan T.S., Alexander S. (2013). Synthesis and characterization of vinyl-functionalized multiwalled carbon nanotubes based molecular imprinted polymer for the separation of chlorpyrifos from aqueous solutions. J. Chem. Technol. Biotechnol..

[148] Lee W.J., Blair A., Hoppin J.A., Lubin J.H., Rusiecki J.A., Sandler D.P., Dosemeci M., Alavanja M.C. (2004). Cancer incidence among pesticide applicators exposed to chlorpyrifos in the Agricultural Health Study. J. Natl. Cancer Inst..

[149] Zhao B., Feng S., Hu Y., Wang S., Lu X. (2019). Rapid determination of atrazine in apple juice using molecularly imprinted polymers coupled with gold nanoparticles-colorimetric/SERS dual chemosensor. Food Chem..

[150] Hayes T., Haston K., Tsui M., Hoang A., Haeffele C., Vonk A. (2002). Feminization of male frogs in the wild. Nature.

[151] Huang C., Cheng Y., Gao Z., Zhang H., Wei J. (2018). Portable label-free inverse opal photonic hydrogel particles serve as facile pesticides colorimetric monitoring. Sens. Actuators B Chem..

[152] Zhang Y., Feng Y.-S., Ren X.-H., He X.-W., Li W.-Y., Zhang Y.-K. (2022). Bimetallic molecularly imprinted nanozyme: Dual-mode detection platform. Biosens. Bioelectron..

[153] Deng H., Wang B., Wu M., Deng B., Xie L., Guo Y. (2019). Rapidly colorimetric detection of caffeine in beverages by silver nanoparticle sensors coupled with magnetic molecularly imprinted polymeric microspheres. Int. J. Food Sci. Technol..

[154] Guo X., Yao S., Li H., Shi X., Pang B., Jin J., Su Z., Zhang H., Zhao C., Wang J. (2021). Multi-functional magnetic molecular imprinting probe for visual detection of IgY antibodies. Microchim. Acta.

[155] Sawetwong P., Chairam S., Jarujamrus P., Amatatongchai M. (2021). Enhanced selectivity and sensitivity for colorimetric determination of glyphosate using Mn–ZnS quantum dot embedded molecularly imprinted polymers combined with a 3D-microfluidic paper-based analytical device. Talanta.

[156] Zhang Y., Jin Z., Zeng Q., Huang Y., Gu H., He J., Liu Y., Chen S., Sun H., Lai J. (2019). Visual test for the presence of the illegal additive ethyl anthranilate by using a photonic crystal test strip. Microchim. Acta.

[157] Lu W., Dong X., Qiu L., Yan Z., Meng Z., Xue M., He X., Liu X. (2017). Colorimetric sensor arrays based on pattern recognition for the detection of nitroaromatic molecules. J. Hazard. Mater..

[158] Liu B., Zhu H., Feng R., Wang M., Hu P., Pan J., Niu X. (2022). Facile molecular imprinting on magnetic nanozyme surface for highly selective colorimetric detection of tetracycline. Sens. Actuators B Chem..

[159] Xu L., Zhang H., Xiong P., Zhu Q., Liao C., Jiang G. (2021). Occurrence, fate, and risk assessment of typical tetracycline antibiotics in the aquatic environment: A review. Sci. Total Environ..

[160] Xu J., Shang M., Liu J., Chen X., Cao Y. (2021). Simultaneous self-assembly of molecularly imprinted magnetic nanoparticles to construct a magnetically responsive photonic crystals sensor for bisphenol A. Sens. Actuators B Chem..

[161] Zeng L., Cui H., Chao J., Huang K., Wang X., Zhou Y., Jing T. (2020). Colorimetric determination of tetrabromobisphenol A based on enzyme-mimicking activity and molecular recognition of metal-organic framework-based molecularly imprinted polymers. Microchim. Acta.

[162] Ye T., Yin W., Zhu N., Yuan M., Cao H., Yu J., Gou Z., Wang X., Zhu H., Reyihanguli A. (2018). Colorimetric detection of pyrethroid metabolite by using surface molecularly imprinted polymer. Sens. Actuators B Chem..

[163] Wu Z., He D., Cui B., Jin Z. (2019). Ultrasensitive detection of microcystin-LR with gold immunochromatographic assay assisted by a molecular imprinting technique. Food Chem..

[164] Tang L., Liang K., Wang L., Chen C., Cai C., Gong H. (2022). Construction of an Ultrasensitive Molecularly Imprinted Virus Sensor Based on an “Explosive” Secondary Amplification Strategy for the Visual Detection of Viruses. Anal. Chem..

[165] Owe-Young R., Webster N.L., Mukhtar M., Pomerantz R.J., Smythe G., Walker D., Armati P.J., Crowe S.M., Brew B.J. (2008). Kynurenine pathway metabolism in human blood–brain–barrier cells: Implications for immune tolerance & neurotoxicity. J. Neurochem..

[166] Rizvi A.S., Murtaza G., Yan D., Irfan M., Xue M., Meng Z.H., Qu F. (2020). Development of molecularly imprinted 2D photonic crystal hydrogel sensor for detection of L-Kynurenine in human serum. Talanta.

[167] Wang H., Wang J., Wang Y., Liu Y., Liu R., Wang X., Tan H., Wang T., Kong T. (2020). Oriented boronate affinity–imprinted inverse opal hydrogel for glycoprotein assay via colorimetry. Microchim. Acta.

[168] Shen M., Wang Y., Kan X. (2021). Dual-recognition colorimetric sensing of thrombin based on surface-imprinted aptamer–Fe_3_O_4_. J. Mater. Chem. B.

[169] Torrini F., Caponi L., Bertolini A., Palladino P., Cipolli F., Saba A., Paolicchi A., Scarano S., Minunni M. (2022). A biomimetic enzyme-linked immunosorbent assay (BELISA) for the analysis of gonadorelin by using molecularly imprinted polymer-coated microplates. Anal. Bioanal. Chem..

[170] Chen W., Fu M., Zhu X., Liu Q. (2019). A close-packed imprinted colloidal array for naked-eye detection of glycoproteins under physiological pH. Biosens. Bioelectron..

[171] Chen T., Zhang A., Cheng Y., Zhang Y., Fu D., Liu M., Li A., Liu J. (2021). A molecularly imprinted nanoreactor with spatially confined effect fabricated with nano-caged cascaded enzymatic system for specific detection of monosaccharides. Biosens. Bioelectron..

[172] Kantiani L., Farré M., Barceló D. (2009). Analytical methodologies for the detection of β-lactam antibiotics in milk and feed samples. TrAC Trends Anal. Chem..

[173] Lowdon J.W., Diliën H., van Grinsven B., Eersels K., Cleij T.J. (2021). Colorimetric sensing of amoxicillin facilitated by molecularly imprinted polymers. Polymers.

[174] Lowdon J.W., Eersels K., Rogosic R., Heidt B., Dilien H., Redeker E.S., Peeters M., van Grinsven B., Cleij T.J. (2019). Substrate displacement colorimetry for the detection of diarylethylamines. Sens. Actuators B Chem..

[175] Karim K., Lamaoui A., Amine A. (2022). Acetazolamide smartphone-based detection via its competition with sulfamethoxazole on molecularly imprinted polymer: A proof-of-concept. J. Pharm. Biomed. Anal..

[176] Kadhim R.A., Abdul A.K.K., Yuan L. (2022). Advances in surface plasmon resonance-based plastic optical fiber sensors. IETE Tech. Rev..

[177] Cennamo N., Pesavento M., Profumo A., Merli D., De Maria L., Chemelli C., Zeni L. Chemical sensors based on surface plasmon resonance in a plastic optical fiber for multianalyte detection in oil-filled power transformer. Proceedings of the Third National Conference on Sensors.

[178] Gandhi M.A., Chu S., Senthilnathan K., Babu P.R., Nakkeeran K., Li Q. (2019). Recent advances in plasmonic sensor-based fiber optic probes for biological applications. Appl. Sci..

[179] Maier S.A. (2007). Plasmonics: Fundamentals and Applications.

[180] Liu J., Jalali M., Mahshid S., Wachsmann-Hogiu S. (2020). Are plasmonic optical biosensors ready for use in point-of-need applications?. Analyst.

[181] Daghestani H.N., Day B.W. (2010). Theory and applications of surface plasmon resonance, resonant mirror, resonant waveguide grating, and dual polarization interferometry biosensors. Sensors.

[182] Chen Y., Ming H. (2012). Review of surface plasmon resonance and localized surface plasmon resonance sensor. Photonic Sens..

[183] Esen C., Piletsky S.A. (2021). Surface Plasmon Resonance Sensors Based on Molecularly Imprinted Polymers. Plasmonic Sensors and their Applications.

[184] Mayer K.M., Hafner J.H. (2011). Localized surface plasmon resonance sensors. Chem. Rev..

[185] Verellen N., Van Dorpe P., Huang C., Lodewijks K., Vandenbosch G.A., Lagae L., Moshchalkov V.V. (2011). Plasmon line shaping using nanocrosses for high sensitivity localized surface plasmon resonance sensing. Nano Lett..

[186] Cennamo N., D’Agostino G., Porto G., Biasiolo A., Perri C., Arcadio F., Zeni L. (2018). A molecularly imprinted polymer on a plasmonic plastic optical fiber to detect perfluorinated compounds in water. Sensors.

[187] Yılmaz E., Özgür E., Bereli N., Türkmen D., Denizli A. (2017). Plastic antibody based surface plasmon resonance nanosensors for selective atrazine detection. Mater. Sci. Eng. C.

[188] Pesavento M., Zeni L., De Maria L., Alberti G., Cennamo N. (2021). SPR-optical fiber-molecularly imprinted polymer sensor for the detection of furfural in wine. Biosensors.

[189] Ayankojo A.G., Reut J., Öpik A., Furchner A., Syritski V. (2018). Hybrid molecularly imprinted polymer for amoxicillin detection. Biosens. Bioelectron..

[190] Cennamo N., D’Agostino G., Perri C., Arcadio F., Chiaretti G., Parisio E.M., Camarlinghi G., Vettori C., Di Marzo F., Cennamo R. (2021). Proof of concept for a quick and highly sensitive on-site detection of SARS-CoV-2 by plasmonic optical fibers and molecularly imprinted polymers. Sensors.

[191] Ertürk Bergdahl G., Andersson T., Allhorn M., Yngman S., Timm R., Lood R. (2019). In vivo detection and absolute quantification of a secreted bacterial factor from skin using molecularly imprinted polymers in a surface plasmon resonance biosensor for improved diagnostic abilities. ACS Sens..

[192] Alberti G., Zanoni C., Spina S., Magnaghi L.R., Biesuz R. (2023). Trends in Molecularly Imprinted Polymers (MIPs)-Based Plasmonic Sensors. Chemosensors.

[193] Walcarius A., Collinson M.M. (2009). Analytical chemistry with silica sol-gels: Traditional routes to new materials for chemical analysis. Annu. Rev. Anal. Chem..

[194] Cennamo N., D’Agostino G., Galatus R., Bibbò L., Pesavento M., Zeni L. (2013). Sensors based on surface plasmon resonance in a plastic optical fiber for the detection of trinitrotoluene. Sens. Actuators B Chem..

[195] Cennamo N., Pesavento M., Lunelli L., Vanzetti L., Pederzolli C., Zeni L., Pasquardini L. (2015). An easy way to realize SPR aptasensor: A multimode plastic optical fiber platform for cancer biomarkers detection. Talanta.

[196] Cennamo N., Zeni L., Tortora P., Regonesi M.E., Giusti A., Staiano M., D’Auria S., Varriale A. (2018). A High Sensitivity Biosensor to detect the presence of perfluorinated compounds in environment. Talanta.

[197] Saito K., Uemura E., Ishizaki A., Kataoka H. (2010). Determination of perfluorooctanoic acid and perfluorooctane sulfonate by automated in-tube solid-phase microextraction coupled with liquid chromatography–mass spectrometry. Anal. Chim. Acta.

[198] Scott B.F., Moody C.A., Spencer C., Small J.M., Muir D.C., Mabury S.A. (2006). Analysis for perfluorocarboxylic acids/anions in surface waters and precipitation using GC− MS and analysis of PFOA from large-volume samples. Environ. Sci. Technol..

[199] Liang J., Deng X., Tan K. (2015). An eosin Y-based “turn-on” fluorescent sensor for detection of perfluorooctane sulfonate. Spectrochim. Acta Part A Mol. Biomol. Spectrosc..

[200] Clark R.B., Dick J.E. (2020). Electrochemical sensing of perfluorooctanesulfonate (PFOS) using ambient oxygen in river water. ACS Sens..

[201] Pitruzzella R., Arcadio F., Perri C., Del Prete D., Porto G., Zeni L., Cennamo N. (2023). Ultra-Low Detection of Perfluorooctanoic Acid Using a Novel Plasmonic Sensing Approach Combined with Molecularly Imprinted Polymers. Chemosensors.

[202] Al Amin M., Sobhani Z., Liu Y., Dharmaraja R., Chadalavada S., Naidu R., Chalker J.M., Fang C. (2020). Recent advances in the analysis of per-and polyfluoroalkyl substances (PFAS)—A review. Environ. Technol. Innov..

[203] Corsini E., Sangiovanni E., Avogadro A., Galbiati V., Viviani B., Marinovich M., Galli C.L., Dell’Agli M., Germolec D.R. (2012). In vitro characterization of the immunotoxic potential of several perfluorinated compounds (PFCs). Toxicol. Appl. Pharmacol..

[204] Culver H.R., Wechsler M.E., Peppas N.A. (2018). Label-free detection of tear biomarkers using hydrogel-coated gold nanoshells in a localized surface plasmon resonance-based biosensor. ACS Nano.

[205] Giljohann D.A., Mirkin C.A. (2009). Drivers of biodiagnostic development. Nature.

[206] Guerreiro J.R.L., Teixeira N., De Freitas V., Sales M.G.F., Sutherland D.S. (2017). A saliva molecular imprinted localized surface plasmon resonance biosensor for wine astringency estimation. Food Chem..

[207] Obreque-Slier E., López-Solís R., Peña-Neira Á., Zamora-Marín F. (2010). Tannin–protein interaction is more closely associated with astringency than tannin–protein precipitation: Experience with two oenological tannins and a gelatin. Int. J. Food Sci. Technol..

[208] Sharma B., Frontiera R.R., Henry A.-I., Ringe E., Van Duyne R.P. (2012). SERS: Materials, applications, and the future. Mater. Today.

[209] Zhou H., Li X., Wang L., Liang Y., Jialading A., Wang Z., Zhang J. (2021). Application of SERS quantitative analysis method in food safety detection. Rev. Anal. Chem..

[210] Boginskaya I., Gainutdinova A., Gusev A., Mailyan K., Mikhailitsyn A., Sedova M., Vdovichenko A., Ryzhikov I., Chvalun S., Lagarkov A. (2020). Detection of Organic Substances by a SERS Method Using a Special Ag-Poly(Chloro-P-Xylylene)-Ag Sandwich Substrate. Coatings.

[211] Guo X., Li J., Arabi M., Wang X., Wang Y., Chen L. (2020). Molecular-Imprinting-Based Surface-Enhanced Raman Scattering Sensors. ACS Sens..

[212] Ma J., Yan M., Feng G., Ying Y., Chen G., Shao Y., She Y., Wang M., Sun J., Zheng L. (2021). An overview on molecular imprinted polymers combined with surface-enhanced Raman spectroscopy chemical sensors toward analytical applications. Talanta.

[213] Hua M.Z., Feng S., Wang S., Lu X. (2018). Rapid detection and quantification of 2,4-dichlorophenoxyacetic acid in milk using molecularly imprinted polymers–surface-enhanced Raman spectroscopy. Food Chem..

[214] Lin X., Wang Y., Wang L., Lu Y., Li J., Lu D., Zhou T., Huang Z., Huang J., Huang H. (2019). Interference-free and high precision biosensor based on surface enhanced Raman spectroscopy integrated with surface molecularly imprinted polymer technology for tumor biomarker detection in human blood. Biosens. Bioelectron..

[215] Zhang Y., Huang Y., Kang Y., Miao J., Lai K. (2021). Selective recognition and determination of malachite green in fish muscles via surface-enhanced Raman scattering coupled with molecularly imprinted polymers. Food Control.

[216] Jameson D.M. (2014). Introduction to Fluorescence.

[217] Turiel E., Martín-Esteban A. (2019). Molecularly imprinted polymers-based microextraction techniques. TrAC Trends Anal. Chem..

[218] Dabrowski M., Lach P., Cieplak M., Kutner W. (2018). Nanostructured molecularly imprinted polymers for protein chemosensing. Biosens. Bioelectron..

[219] Ansari S., Masoum S. (2021). Recent advances and future trends on molecularly imprinted polymer-based fluorescence sensors with luminescent carbon dots. Talanta.

[220] Duan N., Chen X., Lin X., Ying D., Wang Z., Yuan W., Wu S. (2023). based fluorometric sensing of malachite green using synergistic recognition of aptamer-molecularly imprinted polymers and luminescent metal–organic frameworks. Sens. Actuators B Chem..

[221] Quiñone D., Belluzzi M., Torres J., Brovetto M., Veiga N. (2022). Fluoride-selective chemosensor based on an anion imprinted fluorescent polymer. Polyhedron.

[222] Kaminsky L.S., Mahoney M.C., Leach J., Melius J., Jo Miller M. (1990). Fluoride: Benefits and risks of exposure. Crit. Rev. Oral Biol. Med..

[223] Li M., Liu Z., Wang H.-C., Sedgwick A.C., Gardiner J.E., Bull S.D., Xiao H.-N., James T.D. (2018). Dual-function cellulose composites for fluorescence detection and removal of fluoride. Dye. Pigment..

[224] Karrat A., Palacios-Santander J.M., Amine A., Cubillana-Aguilera L. (2022). A novel magnetic molecularly imprinted polymer for selective extraction and determination of quercetin in plant samples. Anal. Chim. Acta.

[225] Lesjak M., Beara I., Simin N., Pintać D., Majkić T., Bekvalac K., Orčić D., Mimica-Dukić N. (2018). Antioxidant and anti-inflammatory activities of quercetin and its derivatives. J. Funct. Foods.

[226] Chen Y., Fan F., Fang G., Deng Q., Wang S. (2020). Fluorometric determination of tyramine by molecularly imprinted upconversion fluorescence test strip. Microchim. Acta.

[227] Alizadeh N., Kamalabadi M., Mohammadi A. (2017). Determination of histamine and tyramine in canned fish samples by headspace solid-phase microextraction based on a nanostructured polypyrrole fiber followed by ion mobility spectrometry. Food Anal. Methods.

[228] Chen X., Liu Y., Li P., Xing Y., Huang C. (2021). Molecularly imprinted silica-coated CdTe quantum dots for fluorometric determination of trace chloramphenicol. Molecules.

[229] Qi Z., Lu R., Wang S., Xiang C., Xie C., Zheng M., Tian X., Xu X. (2021). Selective fluorometric determination of microcystin-LR using a segment template molecularly imprinted by polymer-capped carbon quantum dots. Microchem. J..

[230] Raksawong P., Nurerk P., Chullasat K., Kanatharana P., Bunkoed O. (2019). A polypyrrole doped with fluorescent CdTe quantum dots and incorporated into molecularly imprinted silica for fluorometric determination of ampicillin. Microchim. Acta.

[231] Wackerlig J., Lieberzeit P.A. (2015). Molecularly imprinted polymer nanoparticles in chemical sensing—Synthesis, characterisation and application. Sens. Actuators B Chem..

[232] BelBruno J.J. (2019). Molecularly Imprinted Polymers. Chem. Rev..

[233] Malik M.I., Shaikh H., Mustafa G., Bhanger M.I. (2019). Recent Applications of Molecularly Imprinted Polymers in Analytical Chemistry. Sep. Purif. Rev..

[234] Saylan Y., Akgönüllü S., Yavuz H., Ünal S., Denizli A. (2019). Molecularly Imprinted Polymer Based Sensors for Medical Applications. Sensors.

[235] Vasapollo G., Sole R.D., Mergola L., Lazzoi M.R., Scardino A., Scorrano S., Mele G. (2011). Molecularly Imprinted Polymers: Present and Future Prospective. Int. J. Mol. Sci..

